# Characterization of the antagonistic potential of the glyphosate-tolerant *Pseudomonas resinovorans* SZMC 25872 strain against the plant pathogenic bacterium *Agrobacterium tumefaciens*

**DOI:** 10.3389/fpls.2022.1034237

**Published:** 2022-11-28

**Authors:** Anuar R. Zhumakayev, Mónika Varga, Mónika Vörös, Sándor Kocsubé, Pramod W. Ramteke, András Szekeres, Csaba Vágvölgyi, Lóránt Hatvani, Tamás Marik

**Affiliations:** ^1^ Department of Microbiology, Faculty of Science and Informatics, University of Szeged, Szeged, Hungary; ^2^ ELKH-SZTE Fungal Pathogenicity Mechanisms Research Group, University of Szeged, Szeged, Hungary; ^3^ Department of Biotechnology, Dr. Ambedkar College, Deekshbhoomi, Nagpur, India

**Keywords:** *Pseudomonas resinovorans*, *Agrobacterium tumefaciens*, antagonism, siderophores, extracellular enzymes, novel bioactive metabolites, glyphosate-tolerant bacteria

## Abstract

The utilization of microorganisms with biocontrol activity against fungal and bacterial pathogens of plants is recognized as a promising, effective, and environment-friendly strategy to protect agricultural crops. We report the glyphosate-tolerant *Pseudomonas resinovorans* SZMC 25872 isolate as a novel strain with antagonistic potential towards the plant pathogenic bacterium *Agrobacterium tumefaciens*. In our studies, the growth of the *P. resinovorans* SZMC 25872 and *A. tumefaciens* SZMC 14557 isolates in the presence of 74 different carbon sources, and the effect of 11 carbon sources utilized by both strains on the biocontrol efficacy was examined. Seven variations of media with different carbon sources were selected for the assays to observe the biocontrol potential of the *P. resinovorans* strain. Also, 50% concentrations of the cell-free culture filtrates (CCF) obtained from medium amended with L-alanine or succinic acid as sole carbon source were found to be effective for the growth suppression of *A. tumefaciens* by 83.03 and 56.80%, respectively. The effect of 7 media on siderophore amount and the activity of extracellular trypsin- and chymotrypsin-like proteases, as well as esterases were also evaluated. Significant positive correlation was found between the siderophore amount and the percentage of inhibition, and the inhibitory effect of the CCFs obtained from medium amended with succinic acid was eliminated in the presence of an additional iron source, suggesting that siderophores produced by *P. resinovorans* play an important role in its antagonistic potential. The metabolic profile analysis of the *P. resinovorans* SZMC 25872 strain, performed by high performance liquid chromatography - high resolution mass spectrometry (HPLC-HRMS), has identified several previously not reported metabolites that might play role in the antagonistic effect against *A. tumefaciens*. Based on our findings we suggest that the possible inhibition modes of *A. tumefaciens* SZMC 14557 by *P. resinovorans* SZMC 25872 include siderophore-mediated suppression, extracellular enzyme activities and novel bioactive metabolites.

## 1 Introduction

Modern pest management, aiming the prevention of potential crop losses, is mainly based on the usage of agrochemicals or synthetic pesticides (Syed Ab Rahman et al., 2018). Global agrochemical market was reported to be 37.9 billion USD in 2009 (Glare et al., 2012). However, the continuous growth of pesticide usage (Carvalho, 2006) has resulted in the development of fungicide (Syed Ab Rahman et al., 2018) and herbicide (Hicks et al., 2018) resistance of different pathogens and pests, while the application of higher dosage of the chemicals to overcome resistance is causing environmental pollution and human health problems (Carvalho, 2006; Thakore, 2006). For example, the detrimental effect of the most popular herbicide glyphosate (Benbrook, 2016), on different soil-inhabiting organisms including bacteria, fungi, and earthworms has been widely reported (Huber et al., 2005; Gaupp-Berghausen et al., 2015; Druille et al., 2016). The exposure to glyphosate has led to changes in the gut microbiota of different organisms, such as bees and cows (Krüger et al., 2013; Van Bruggen et al., 2018; Motta et al., 2018; Rueda-Ruzafa et al., 2019). Furthermore, glyphosate was reported to have the detrimental effect on the human lymphocytes and buccal epithelial cells (Lioi et al., 1998; Koller et al., 2012), and it was included in the group of potentially carcinogenic substances by the International Agency for Research on Cancer (IARC, 2015; Tarazona et al., 2017). Therefore, ecologically safe approaches have raised public and scientific interest for both the control of agricultural pests and the prevention of the contamination of food, soil, and ecosystems. Consequently, it has resulted in the development of various alternative means of pest management, such as the use of biopesticides, including the application of microbial species possessing antagonistic activity, by which effective pest control can be achieved in an environment-friendly manner (Mnif and Ghribi, 2015; Syed Ab Rahman et al., 2018).

The genus *Agrobacterium* consists of Gram-negative bacteria with the ability of horizontal gene transfer with their host plants (Subramoni et al., 2014). Agrobacteria are common inhabitants of soils, associated with roots, tubers, and underground steams of plants (Matthysse, 2005). Among the different species, *A. tumefaciens* and *A. vitis* are the most recognized species known to cause crown-gall formation in plants. Crown-gall disease is spread worldwide and causes remarkable economic losses, particularly in grape cultivations (Filo et al., 2013). *A. tumefaciens* possesses a serious challenge in pest management as it can infect 93 families, 331 genera, and 643 species including both monocotyledonous and dicotyledonous plants (Conner and Dommisse, 1992; Xie et al., 2021). The disease causes plant growth inhibition and yield losses, furthermore, severe cases can result in plant death (Filo et al., 2013; Zhou et al., 2020). Other plants can also be easily infected; therefore, disease management represents a serious concern (Dandurishvili et al., 2011). Currently, farmers apply chemical and physical methods to control crown-gall disease. In addition to the prevention measures (selection of pathogen-free seedling material, graft union protection, renewal of trunk) and chemical methods (fumigation and solarization), Filo et al. (2013) proposed biological control as the most efficient technique to control crown-gall disease. Strains of a variety of microbial species, such as *Pseudomonas fluorescens, Bacillus amyloliquefaciens*, *B. subtilis*, and *Phomopsis liquidambari* were also reported to be effective for the biological control of *A. tumefaciens* (Hammami et al., 2008; Dandurishvili et al., 2011; Abdallah et al., 2018a; Zhou et al., 2020).

*Bacillus* and *Pseudomonas*-based biopesticides are the most proven biocontrol agents. The genus *Pseudomonas* involves Gram-negative, motile, aerobic bacteria, representing one of the most heterogeneous and ecologically significant groups among all known bacterial taxa (Mnif and Ghribi, 2015). Some species, such as *P. syringae* or *P. aeruginosa* are serious plant (Xin et al., 2018) or human (Pang et al., 2019) pathogens, respectively, while other members of the genus, including *P. fluorescences*, *P. putida*, and *P. chlororaphis* are well-recognized agents for biocontrol, plant growth promotion, and bioremediation (Mnif and Ghribi, 2015; He et al., 2019; Setlhare et al., 2019). The extract of the culture supernatant of the *P. chlororaphis* ssp. *aureofaciens* DSM 6698 strain fully inhibited the growth of *Rhizobium solani*, *Pythium ultimum*, and *Fusarium oxysporum* (Mezaache-Aichour et al., 2013). The volatile organic compounds produced by the tested *P. fluorescens* B-4117 and Q8r1-96 strains inhibited the growth of *A. tumefaciens* and *A. vitis in vitro* (Dandurishvili et al., 2011).

Biocontrol microorganisms apply different tools to limit the growth of plant pathogens, including siderophores, extracellular enzymes, and bioactive metabolites. The siderophore-mediated suppression of plant pathogens was initially suggested in the 1970’s, while the first documented evidence of this mechanism was provided by the studies of Kloepper et al. (1980). One of the first detailed studies of siderophore-mediated suppression of bacterial growth was reported by O’Sullivan and O’Gara (1992). The main mechanism involved in this process is the competition for the limited available iron between the plant pathogens and the beneficial agents, in which the production of siderophores by the latter causes iron-depleted circumstances for the pathogen (O’Sullivan and O’Gara, 1992; Saha et al., 2016). The inhibitory effect of siderophores on various bacterial and fungal plant pathogens has been widely studied (O’Sullivan and O’Gara, 1992; Ambrosi et al., 2000; Mishra and Arora, 2012; Sulochana et al., 2014; Michavila et al., 2017; Tao et al., 2020). Microbial enzymes were also reported to play role in the suppression of plant pathogens, and indirectly stimulate plant growth (Mishra et al., 2020). Extracellular proteases, cellulases, β-1,3-glucanases, chitinases, and lipases are the most important enzymes for the biocontrol agents, as they can be applied by the biocontrol strains directly against fungal pathogens, or in the competition for nutrients and the space in the rhizosphere (Vörös et al., 2019).

Overall, the efficiency of pathogen control, eco-friendly mode of action, various inhibition mechanisms limiting the development of tolerance in pests, lower costs of development, and further beneficial characteristics to improve plant growth and soil quality have raised the governmental, public and industrial interests, which can be expected to expand the prospective of biopesticides for an effective and environment-friendly pest management (Chandler et al., 2011; Glare et al., 2012; Mnif and Ghribi, 2015; Syed Ab Rahman et al., 2018). Importantly, as glyphosate has been the most popular, globally used herbicide since 1974 (Duke and Powles, 2008), it is apparent that a broad range of agricultural soils have become polluted by it and other synthetic agrochemicals, which might have the inhibitory effect on microorganisms, thus beneficial microbial strains to be introduced into agricultural soil need to be tested for their pesticide tolerance in advance. Therefore, the objective of the presented study was the isolation of novel glyphosate-tolerant species possessing antagonistic activity towards *A. tumefaciens*, and the examination of the possible inhibition mechanisms involved in the biocontrol potential.

## 2 Materials and methods

### 2. 1 Bacterial strains and culture conditions

Glyphosate-tolerant bacteria were isolated, and identified based on the sequence analysis of fragments of their 16S rRNA and *rpoB* genes in our previous study (Zhumakayev et al., 2021). Briefly, 5 g of soil sample previously exposed to the glyphosate-containing herbicide “Glifol” (Galenika Fitofarmacija, Belgrade, Serbia) was suspended in 50 ml sterile physiological saline solution (0.9% NaCl in distilled water). Three enrichment-culture techniques, in which 0.1 or 1 mg/ml glyphosate (*Glialka Star*, Monsanto Europe, Brussels, Belgium; active ingredient: glyphosate, 360 g/l) served as the sole carbon and nitrogen source, were applied to recover glyphosate-tolerant bacteria. The growth of the isolated strains was evaluated on Solid Minimal Medium (SMM: 1 g/l KH_2_PO_4_, 3 g/l Na_2_HPO_4_, 1 g/l MgSO_4_·7H_2_O, 20 g/l agarose, Hatvani et al., 2013) supplemented with 1 mg/ml glyphosate and 10 isolates possessing the most intensive growth as well as different colony morphology, considered as glyphosate-tolerant strains, were selected for the subsequent studies. The list of glyphosate-tolerant isolates and *Agrobacterium* strains involved in antagonistic activity assays are listed in **Table 1**.

**Table 1:** List of the studied glyphosate-tolerant isolates and *Agrobacterium* strains.

Species	SZMC* number
Glyphosate-tolerant bacteria	
*Pseudomonas resinovorans* (genotype II)	SZMC 25848
*P. resinovorans* (genotype II)	SZMC 25851
*Ensifer adhaerens*	SZMC 25856
*P. resinovorans* (genotype II)	SZMC 25859
*P. resinovorans* (genotype I)	SZMC 25863
*P. resinovorans* (genotype I)	SZMC 25870
*E. adhaerens*	SZMC 25871
*P. resinovorans* (genotype I)	SZMC 25872
*P. resinovorans* (genotype II)	SZMC 25875
*Agrobacterium* strains	
*Agrobacterium tumefaciens*	SZMC 14554, SZMC 14555, SZMC 14556, SZMC 14557, SZMC 21395, SZMC 21407, and SZMC 21783
*A. vitis*.	SZMC 21396, SZMC 21397, SZMC 21398, SZMC 21784, SZMC 21785, SZMC 21786, and SZMC 21787

*SZMC: Szeged Microbiology Collection.

The bacterial isolates were maintained on 39 g/l Potato Dextrose Agar (PDA) (Wise et al., 2006) at 25°C. Chemicals and reagents unless specified were purchased from Sigma-Aldrich, Merck Ltd. (Budapest, Hungary).

Multiple alignments of the obtained sequences of the *Pseudomonas resinovorans* isolates were conducted using MAFFT v. 7.453 (Katoh and Standley, 2013) with the E-INS-i iterative refinement method. The alignments were concatenated and subjected for phylogenetic inference. Phylogenetic reconstruction was carried out by using IQ-TREE v. 1.6.12 (Nguyen et al., 2015) with the K2P+G4 model for the 16S and HKY+F+G4 model for the *rpob* dataset determined by the inbuilt ModelFinder tool (Kalyaanamoorthy et al., 2017). Statistical support of the best tree was calculated with Ultrafast bootstrap approximation (Hoang et al., 2018) with 5000 replicates.

### 2.2 *In vitro* antagonistic activity assays

Dual-culture method was applied for the determination of the potential antagonistic activity of the glyphosate-tolerant bacteria against pathogenic *Agrobacterium* sp. isolates, which were obtained from the Szeged Microbiology Collection (SZMC, http://www.wfcc.info/ccinfo/collection/by_id/987). Fresh cultures pre-grown for 24 h on PDA were used to prepare bacterial suspensions in sterile physiological saline solution (0.9% NaCl) at 10^6^ and 10^7^ CFU/ml in the case of the *Agrobacterium* and glyphosate-tolerant isolates, respectively. PDA plates were flooded with 5 ml of the *Agrobacterium* suspensions of strains to cover the full surface. The remaining suspension was removed, the plates were air-dried for 20 min, then 10 µl suspension of the glyphosate-tolerant isolates were spot-inoculated on the surface (three strains per plate), then the plates were air-dried for further 10 min. Plates inoculated solely with the *Agrobacterium*, or the glyphosate-tolerant isolates served as controls. The inoculated plates were incubated for 3 days at 25°C and monitored for the appearance of halo zones around glyphosate-tolerant strains, which was associated with antagonistic activity. The test was performed in triplicate. The glyphosate-tolerant isolate with the highest inhibition potential and the most vulnerable *A. tumefaciens* strain were selected for the subsequent biocontrol studies.

### 2.3 Carbon source utilization test

*A. tumefaciens* SZMC 14557 and *A. vitis* SZMC 21396 as the most sensitive pathogenic strains and the glyphosate-tolerant bacterial isolate *P. resinovorans* SZMC 25872 showing the highest antagonistic activity were tested for their ability to utilize various compounds as the sole carbon source. Liquid Minimal Medium (LMM: 1 g/l KH_2_PO_4_, 0.5 g/l MgSO_4_·7H_2_O, 5 g/l (NH_4_)_2_SO_4_ in distilled water, Vágvölgyi et al., 1996) was amended individually with 74 different carbon-containing substances as follows: (-) quinic acid (1), 2-keto-D-gluconic acid (2), adenosine (3), alpha-methyl-D-mannoside (4), ascorbic acid (5), beta-alanine (6), beta-methyl-D-galactoside (1-O-methyl-beta-D-galactopyranoside) (7), cellobiose (8), cis-aconitic acid (9), cytidine (10), cytosine (11), D-arabinose (12), dextran (13), D-fructose (14), D-galactose (15), D-glucosamine (16), D-glucose (17), dihydroxyacetone (18), DL-isocitric acid (19), D-lyxose (20), D-mannitol (21), D-mannose (22), ethanol (23), fumaric acid (24), galactitol (25), gallic acid (26), gamma-butyrolactone (27), gentisic acid (28), gluconic acid (29), glycerol (30), glycine (31), i-erythritol (32), inosine (33), inulin (34), ketoisovaleric acid (35), lactose (36), L-alanine (37), L-arabinose (38), L-arginine (39), L-asparagine (40), L-citrulline (41), L-glutamic acid (42), L-glutamine (43), L-histidine (44), L-isoleucine (45), L-lysine (46), L-malic acid (47), L-methionine (48), L-ornithine (49), L-rhamnose (50), L-serine (51), L-sorbose (52), L-threonine (53), L-tryptophan (54), L-valine (55), maltose (56), melezitose (57), melibiose (58), myo-inositol (59), nicotinic acid (60), p-arbutin (61), protocatechuic acid (3,4-dixydroxybenzoic acid) (62), sodium pyruvate (63), raffinose (raffinose pentahydrate) (64), ribitol (adonitol) (65), sorbitol (66), starch (67), succinic acid (68), sucrose (69), thymine (70), uridine (71), vanillin (72), xylan (73), and xylitol 74), which are also listed in **Supplementary Table 1**.

The concentration of each carbon source in LMM was 2 g/l, and the stock solutions of the tested carbon sources were prepared in double concentrated form (4 g/l) in sterile distilled water. For liquid carbon sources (23, 27, and 30), 6 µl of the corresponding compound was dissolved in 1.5 ml sterile distilled water to obtain the desired concentration. The sterility of the prepared stock solutions was checked by inoculating 5 µl amounts on PDA followed by incubation for 3 days at 25°C.

The test was carried out using broth microdilution technique. One-hundred µl sterile LMM was added in wells of a 96-well microtiter plate and mixed with 100 µl of the stock solutions to obtain the final concentration of 2 g/l. Bacterial suspensions were prepared in 20 ml physiological saline solution at 10^6^ CFU/ml, and 20 µl was inoculated in each well to obtain the initial cell concentration 10^5^ CFU/ml. Non-inoculated negative samples amended with 20 µl sterile physiological solution were used to examine any possible increase in OD_620_ due to the tested compounds. The microtiter plates were incubated for 48 h at 25°C, then the optical density (OD_620_) was determined using a spectrophotometer (SPECTROstar Nano microplate reader, BMG Labtech, Offenburg, Germany). Three replicates were applied for each carbon source. Carbon sources utilized by both *P. resinovorans* SZMC 25872 and *A. tumefaciens* SZMC 14557 were selected to test the effect of carbon source on the biocontrol efficacy.

### 2.4 Testing the effect of carbon sources on the antagonistic potential of *P. resinovorans* against *A. tumefaciens*

To study the influence of different carbon sources on the biocontrol activity of the *P. resinovorans* SZMC 25872 isolate against *A. tumefaciens*, solid minimal medium (SMM: 1 g/l KH_2_PO_4_, 0.5 g/l MgSO_4_·7H_2_O, 5 g/l (NH4)_2_SO_4_, 15.0 g/l agar in distilled water, Vágvölgyi et al., 1996) was amended with 11 compounds individually (2 g/l) as the sole carbon source. Based on the results of previous studies (Subsection 2.3), the applied substances were 2, 14, 17, 37, 40, 42, 43, 47, 63, 67, and 68, which were utilized by *P. resinovorans* SZMC 25872 as the most promising antagonist and *A. tumefaciens* SZMC 14557 as the most susceptible strain (Subsection 2.2). The assays were carried out as described in Subsection 2.2. As the initial assays for testing the biocontrol potential of the isolates was performed on PDA (Subsection 2.2), this medium served as the positive control to compare the effect of carbon sources on the growth inhibition of *A. tumefaciens*. The assay was performed in triplicate.

### 2.5 Testing the inhibitory effect of the cell-free culture filtrates of *P. resinovorans* on the growth of *A. tumefaciens*

*P. resinovorans* SZMC 25872 was cultivated in PDB (24 g/l Potato Dextrose Broth), Luria-Bertani (LB: 7.5 g/l peptone, 2.5 g/l NaCl, 2.5 g/l yeast extract, Bertani, 1951) Yeast Extract Glucose (YEG: 2 g/l yeast extract, 2 g/l glucose, Vörös et al., 2019), and LMM (Vágvölgyi et al., 1996) supplied individually with 17, 37, 63, and 68 as sole carbon source, which resulted in the highest biocontrol activity in the previous experiment (Subsection 2.4). Prior to sterilization, the pH of all media was adjusted to 6.8-6.9 using 1 M NaOH, except for PDB, which was used without pH adjustment, and YEG, with its initial pH 6.8. Suspensions of *P. resinovorans* SZMC 25872 (overnight-grown on PDA) were prepared in 5 ml sterile physiological saline solution, which were used to inoculate 50 ml of the different media in 100-ml Erlenmeyer flasks at 10^5^ CFU/ml starting concentration. Non-inoculated flasks containing the same amount of the corresponding media served as the negative controls. The cultures were incubated on a rotary shaking incubator (MaxQ 8000 shaking incubator, Thermo Fisher Scientific, Marietta, OH, USA) at 130 rpm, 28°C for 3 days, and growth was determined based on the cell density (OD_620_) on the final day of incubation. The obtained cultures were transferred to 50-ml sterile Falcon tubes and centrifuged at 8,500 rpm for 10 min (Heraeus Biofuge Primo centrifuge, Thermo Fisher Scientific, Osterode, Germany). Thereafter, approximately 5-7 ml of the obtained supernatants were filtered through 0.22-µm filter membrane (Millex GV, syringe-driven filter unit, Merck, Tullagreen, Carrigtwohill, Cork, Ireland) to remove all remaining bacterial cells. The sterility of the obtained cell-free culture filtrates (CCF) was checked by inoculating 5 µl on PDA, followed by 3-day incubation at 25°C and daily observation for any bacterial growth. The validated CCF samples were stored at -20°C and thawed before use.

Broth microdilution method, using a 96-well microtiter plate was applied to study the inhibitory effect of the obtained CCF samples on the growth of *A. tumefaciens* SZMC 14557. PDB medium was amended with the different CCFs at 50, 25, and 12.5 concentrations, in the total volume of 100 µl. Overnight culture of *A. tumefaciens* grown on PDA was used to prepare suspension in sterile PDB medium at 2×10^6^ CFU/ml concentration, and 100 µl was added to the wells of the microtiter plate already containing the diluted CCFs to obtain the 10^6^ CFU/ml starter cell concentration. Inoculated PDB without CCF and non-inoculated PDB amended with CCFs in the mentioned concentrations served as the positive and negative controls, respectively. The optical density (OD_620_) was measured after 24-h incubation at 25°C. The growth inhibition rate caused by the CCFs was determined as the difference between the positive control and treated samples multiplied by 100% and divided with the value of positive control. The obtained values of the growth inhibition were subjected to correlation analysis (Subsection 2.10) with the data of siderophore amount (OD_630_, Subsection 2.6). CCF samples with the significant inhibition rate (p<0.05) were used for investigating possible modes of action of the growth inhibition of *A. tumefaciens* (Subsections 2.7 and 2.8). The test was carried out in triplicate.

### 2.6 Siderophore production assay

The determination of siderophores in the CCF samples obtained as described in Subsection 2.5 was carried out based on the colorimetric reaction using Chrome Azurol Solution (CAS) (Schwyn and Neilands, 1987). The CAS formulation consisting of the mixture of 3 solutions was prepared as follows: Ten ml FeCl_3_ solution (1 mM FeCl_3_·6H_2_O in 10 mM HCl) was added to 50 ml CAS solution (2 mM CAS in distilled water), then 40 ml HDTMA solution (5 mM hexadecyltrimethylammonium bromide in distilled water) was also added to the mixture. One hundred μl CCF was transferred into the wells of a 96-microtiter plate. Equal amount of CAS solution was added in the wells and the mixtures were homogenized using a pipette. The CAS solution appears as strong, blue-colored compound, which becomes colorless if the siderophores present in the culture supernatant remove Fe^3+^ from the solution. The mixtures were incubated in darkness for 20 min at 25°C and analyzed at OD_630_. The obtained OD_630_ values were used to calculate the percent siderophore unit (psu) according to the following formula:


$$\frac{Ar-As}{Ar}\;*100\;$$

where *Ar* is the absorbance of the reference sample, and *As* is the absorbance of the inoculated sample (Payne, 1994). The assay was performed in triplicate. Correlation analysis between siderophore values and the inhibitory effect was performed as described in Subsection 2.10.

### 2.7 Testing the potential role of siderophores and ROS in the growth suppression of *A. tumefaciens*

CCF samples obtained from LMM amended with L-alanine and succinic acid at the concentrations that caused the most remarkable growth inhibition (25 and 50%) were used to test the possibility of siderophore or reactive oxygen species (ROS)-induced suppression of *A. tumefaciens* SZMC 14557 using the method described earlier (Subsection 2.5). Ten µl FeCl_3_·6H_2_O from 0.58 mg/ml stock solution (final iron concentration in the samples: 29 mg/l, Santos et al., 2014) or 10 µl ascorbic acid from 3.4 mg/ml (final ascorbic acid concentration in samples: 1 mM (0.17 mg/ml), Adler et al., 2012) were added into the treated samples to eliminate possible iron sequestration or ROS-generation, respectively, due to the presence of CCF. Samples containing no additional iron source or ascorbic acid were amended with 10 µl sterile distilled water. All assays were performed in triplicate.

### 2.8 Extracellular enzyme activity assays

Extracellular enzyme activities in the CCF samples of the *P. resinovorans* SZMC 25872 strain grown in different media (Subsection 2.5) were examined using chromogenic substrates (**Table 2**), following the protocol of Vörös et al. (2019).

**Table 2:** Chromogenic substrates used to test extracellular enzyme activities.

Extracellular enzyme system	Substrate
Chymotrypsin-like proteases	*N*-succinyl-L-Ala-L-Ala-L-Pro-L-Phe-p-nitroanilide
Trypsin-like proteases	*N*-Bz-L-Phe-L-Val-L-Arg-p-nitroanilide-hydrochloride
Esterases	p-nitrophenyl-palmitate

The substrates were dissolved in dimethyl sulfoxide to obtain 3 mM concentration, while phosphate buffer (5.7 g/l KH_2_PO_4_, 4.4 g/l Na_2_HPO_4_, pH 6.6) was prepared in distilled water. Fifty µl of the CCF samples and 50 µl phosphate buffer were added in the wells of a 96-well microtiter plate and mixed with 50 µl of the corresponding chromogenic substrate (final concentration 1 mM); then the mixture was incubated for 20 min. The optical density of the reaction mixtures was measured at 405 nm (OD_405_). The test was performed in triplicate.

### 2.9 HPLC-HRMS analysis and identification of the bioactive metabolites potentially involved in the biocontrol of *A. tumefaciens*

To detect and identify the main bioactive compounds, culture supernatant samples of the glyphosate-tolerant *P. resinovorans* SZMC 25872 strain suppressing the growth of *A. tumefaciens* (Subsections 2.5 and 2.7) were subjected to HPLC-HRMS analysis. Liquid cultures were centrifuged at 10,000 *g* for 10 min. Centrifugation was repeated, and 0.5 ml supernatant freeze-dried. Two-hundred μl of 80% methanol was added to the lyophilized residues followed by soaking in an ultrasound bath (80 Hz, 25°C, Elmasonic P, Singen, Germany) for 10 min. Then, samples were stirred up thoroughly with a vortex for 15 sec and kept in ultrasonic bath for 5 more min. After that, samples were homogenized with a brief vortexing and centrifuged (5430R, Eppendorf, Hamburg, Germany) at 18,000 *g* and 20°C for 10 min. Sixty μl of the supernatant was transferred in the HPLC vials and prior to analysis, each sample was amended with 5 μl of the internal standard at 0.1 mg/ml concentration (buspirone, stock at 1 mg/ml dissolved in MilliQ water).

All samples were analyzed in both positive and negative ionization modes of HPLC-HRMS. The measurements were performed using a Dionex Ultimate 3000 UHPLC system (Thermo Fischer Scientific, Waltham, Massachusetts, USA) coupled to a Q Exactive Plus hybrid quadrupole-Orbitrap MS (Thermo Fischer Scientific, Waltham, Massachusetts, USA) operating with a heated electrospray interface (HESI). The metabolites were separated using a Gemini NX C18 3 μm, 150 x 2 mm (Phenomenex, California, USA) column. For the separation of metabolites water (A) and methanol/acetonitrile (B) (1/1, v/v) both supplemented with 0.1% formic acid, served as the mobile phases. The linear gradient was executed as follows: 5% B 0-2 min; increased to 95% B 2-13 min; 95% B until 19 min; 5% B 19-19.5 min and 5% B until 24 min. The flow rate was 0.2 ml/min, the injection volume was 3 µl and the column temperature was maintained at 25°C. The capillary and the heater temperatures were 320 and 300°C, respectively. The sheath gas and the auxiliary gas flow rates were applied at 30 and 10 (in arbitrary units), respectively. The precursor ion scan was done in full MS mode at a resolution of 70,000 at an *m/z* value of 200 (3 scans/s), auto gain control (AGC) target of 3e6, maximum injection time (IT) of 100 ms, and a scan range of *m/z* 100-1500. Isolation window was 0.4 *m/z*. The product ion scan was conducted in a data dependent MS^2^ mode (ddMS^2^) using a resolution of 17,500 at a *m/z* value of 200, AGC target of 2e5, maximum IT of 200 ms, normalized collision energy (NCE) of 30 eV, stepped NCE of 50%. For every full scan, 10 ddMS^2^ scan was carried out. HPLC-HRMS data were acquired using Trace Finder 4.0 software. The raw MS data files were processed using Compound Discoverer™ (2.1) software.

### 2.10 Statistical analysis

Assumptions for parametric tests as normal distribution of the residuals and homoscedasticity (similar variances in all groups) were checked using Shapiro test for normality and QQ-plot, respectively, before applying statistical test. The effect of the CCF samples on the cell density of *A. tumefaciens* SZMC 14557 (OD_620_ data) was analyzed by one-way (ANOVA), followed by post-hoc pair-wise comparisons (Tukey’s test, confidence level was at 95% or p<0.05). The variants not corresponding to the mentioned assumptions for parametric tests were analyzed using non-parametric alternative for ANOVA - Kruskal-Wallis test. The difference between samples treated with and without extra iron source or ascorbic acid was checked with t-test or its non-parametric alternative, Wilcoxon test.

Assumptions, such as linearity and homoscedasticity were checked before correlation analysis; confidence level was set at 95% (p<0.05). Pearson (*r*) coefficient was applied for parametric correlation analysis as the data met the mentioned assumptions. All statistical analyses and graphical data visualization were performed using R (version 3.5.1; https://www.r-project.org/) and the RStudio Desktop software (http://www.rstudio.com/). Package *agricolae* (Mendiburu, 2020) was applied to run Tukey’s test while packages *ggplot2* (Wickham, 2016), *ggpubr* (Kassambara, 2020), and *patchwork* (Pedersen, 2020) were used to prepare the graphs. Unless specified, values throughout the studies are presented as means of three replicates ± standard deviations (SD).

## 3 Results

**Figure 1:**
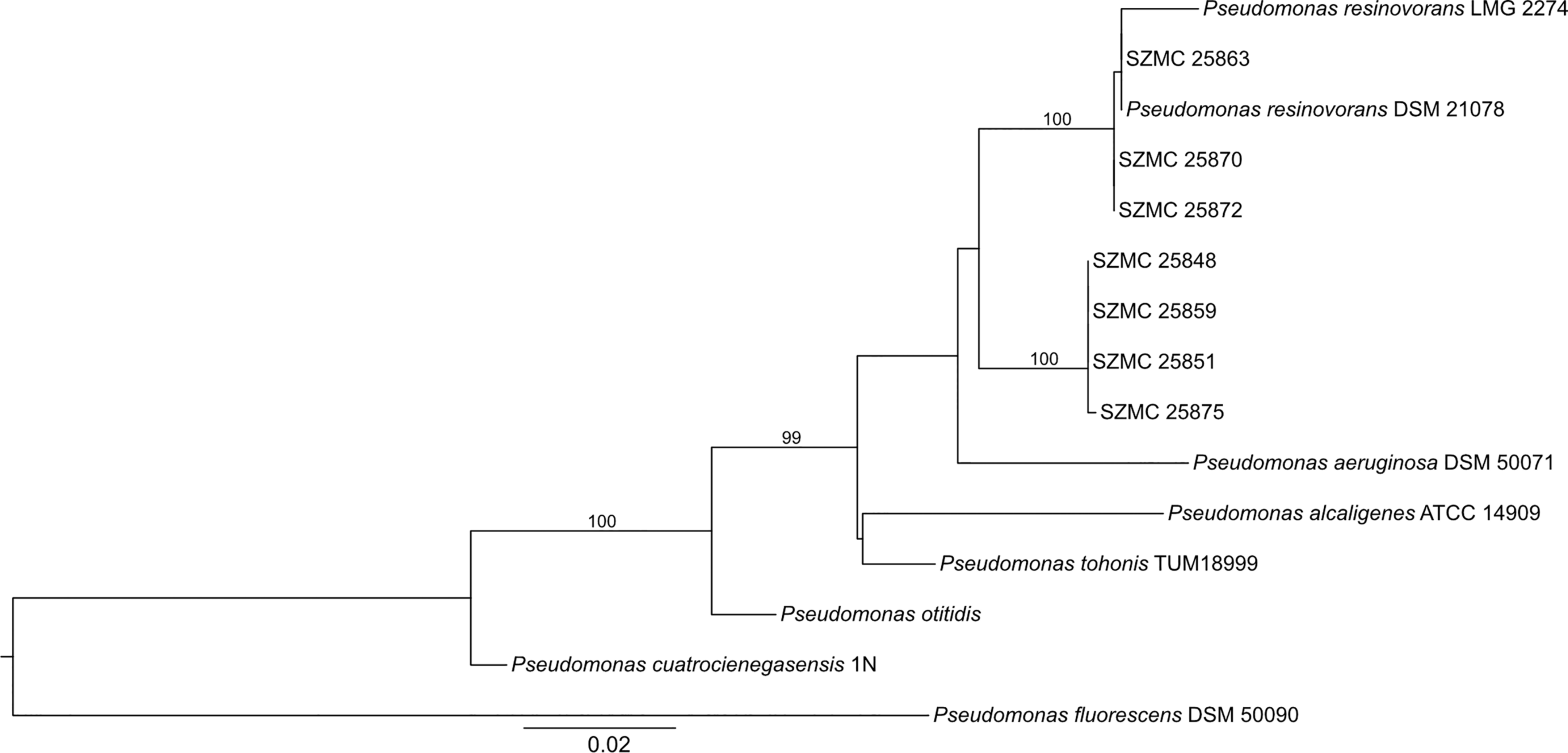
Maximum Likelihood phylogenetic tree of *P. resinovorans* isolates. Statistical support of the best tree was calculated with Ultrafast bootstrap approximation with 5000 replicates. Only bootstrap values above 95% - providing reliable results - are shown.

The phylogenetic analysis revealed that the 7 P*. resinovorans* isolates represent 2 different genotypes (I and II, including 3 and 4 strains, respectively) (**Figure 1**). SZMC 25863, SZMC 25870, and SZMC 25872 were found to be phylogenetically close to *P. resinovorans* DSM 21078 and *P. resinovorans* LMG 2274. The remaining 4 strains (SZMC 25848, SZMC 25851, SZMC 25859, SZMC 25875) belong to *P. resinovorans* genotype II.

### 3.1 *In vitro* antagonistic activity of *P. resinovorans* towards *A. tumefaciens*

All *P. resinovorans* strains of both genotypes (Zhumakayev et al., 2021) were able to substantially inhibit 6 out of the 7 tested *A. tumefaciens* and *A. vitis* (**Table 3**) strains, however, only partial inhibition was observed in the case of *A. vitis*. No antagonistic activity was shown by the two *Ensifer adhaerens* isolates. Single strains of *A. tumefaciens* SZMC 14554 and *A. vitis* SZMC 21784 were not inhibited by any of the *P. resinovorans* isolates, while *A. tumefaciens* SZMC 14557 and *A. vitis* SZMC 21396 were found to be the most susceptible isolates, therefore, they were selected to study their carbon source utilization profiles. Among *P. resinovorans* strains, SZMC 25872 (genotype I, Zhumakayev et al., 2021) showed the most intensive inhibition of *A. tumefaciens* SZMC 14557 and *A. vitis* SZMC 21396 (**Table 3**) and was therefore chosen for detailed characterization in terms of its antagonistic potential in the subsequent assays (Subsection 3.2-3.8).

**Table 3:** Colony diameter (C, mm) and colony + inhibition zone diameter (IZ, mm) values of 7 P*. resinovorans* isolates on susceptible *A. tumefaciens* and *A. vitis* strains.

*P. resinovorans*	*A. tumefaciens*																				
	SZMC 14555			SZMC 14556			SZMC 14557				SZMC 21395				SZMC 21407				SZMC 21783		
	C	IZ		C		IZ	C		IZ		C		IZ		C		IZ		C		IZ
SZMC 25848	10.3 ± 1.2	21.7 ± 0.6		10.0 ± 0.0		27.3 ± 2.5	11.0 ± 1.0		36.3 ± 1.5		10.0 ± 0.0		21.3 ± 3.2		9.7 ± 0.6		22.0 ± 4.0		10.7 ± 1.2		29.0 ± 1.7
SZMC 25851	9.0 ± 0.0	19.7 ± 2.5		10.0 ± 0.0		24.7 ± 2.1	10.3 ± 1.2		32.7 ± 2.1		10.0 ± 0.0		21.7 ± 1.5		9.3 ± 0.6		18.0 ± 2.0		9.7 ± 0.6		27.7 ± 2.5
SZMC 25859	9.3 ± 1.2	21.7 ± 2.1		9.7 ± 1.2		21.0 ± 2.0	10.7 ± 0.6		35.3 ± 2.9		9.0 ± 1.0		18.7 ± 2.5		9.3 ± 0.6		22.0 ± 3.0		10.0 ± 0.0		29.0 ± 3.5
SZMC 25863	8.3 ± 0.6	18.0 ± 1.0		10.0 ± 0.0		17.7 ± 0.6	10.3 ± 0.6		24.3 ± 0.6		9.0 ± 1.0		15.3 ± 0.6		9.7 ± 0.6		16.0 ± 0.0		10.0 ± 0.0		21.0 ± 3.6
SZMC 25870	9.3 ± 0.6	17.7 ± 1.2		10.7 ± 0.6		20.0 ± 1.7	7.7 ± 3.2		22.0 ± 1.0		8.5 ± 0.7		16.5 ± 0.7^b^		9.3 ± 0.6		16.7 ± 0.6		10.0 ± 1.0		20.3 ± 2.1
SZMC 25872	9.3 ± 0.6	19.0 ± 2.0		9.7 ± 0.6		17.0 ± 2.0	10.7 ± 0.6		24.7 ± 1.5		9.0 ± 1.0		16.0 ± 1.0		9.0 ± 1.4		14.5 ± 0.7^b^		9.7 ± 1.2		21.0 ± 1.0
SZMC 25875	9.3 ± 1.2	23.7 ± 3.5		10.3 ± 0.6		19.0 ± 1.7	9.7 ± 0.6		31.3 ± 2.3		9.7 ± 0.6		18.7 ± 1.5		9.7 ± 0.6		21.0 ± 4.4		10.3 ± 0.6		25.7 ± 1.5
***P. resinovorans***	***A. vitis***																				
	SZMC 21396		SZMC 21397				SZMC 21398			SZMC 21785				SZMC 21786				SZMC 21787			
	C	IZ	C		IZ		C	IZ		C		IZ		C		IZ		C		IZ	
SZMC 25848	9.7 ± 0.6	34.7 ± 2.9	10.0 ± 1.0		16.0 ± 0.0^b^		9.3 ± 0.6	27.0 ± 2.0		10.3 ± 0.6		29.3 ± 1.5		10.0 ± 1.0		22.0^c^		10.0 ± 0.0		35.0 ± 1.4^b^	
SZMC 25851	9.3 ± 0.6	33.7 ± 3.1	10.0 ± 1.0		21.5 ± 3.5^b^		9.7 ± 0.6	29.0 ± 2.0		9.7 ± 0.6		29.3 ± 5.0		10.3 ± 0.6		22.0^c^		11.0 ± 1.4		35.5 ± 0.7^b^	
SZMC 25859	9.7 ± 0.6	34.7 ± 5.5	10.3 ± 0.6		10.3 ± 0.6^a^		10.0 ± 1.0	27.0 ± 7.2		10.3 ± 0.6		31.0 ± 1.0		9.3 ± 0.6		26.0 ± 7.1^b^		8.0 ± 0.0		36.0^c^	
SZMC 25863	9.3 ± 0.6	28.0 ± 1.0	10.0 ± 0.0		10.0 ± 0.0^a^		10.7 ± 0.6	21.7 ± 1.5		10.0 ± 1.0		22.3 ± 2.5		10.3 ± 1.2		23.0^c^		10.0 ± 0.0		29.0 ± 4.2^b^	
SZMC 25870	10.3 ± 0.6	28.3 ± 1.5	11.0 ± 1.0		11.0 ± 1.0^a^		9.7 ± 1.2	18.0 ± 2.0		11.0 ± 1.0		23.7 ± 3.1		9.3 ± 0.6		22.0 ± 1.4^b^		10.5 ± 0.7		25.0 ± 7.1^b^	
SZMC 25872	10.0 ± 0.0	28.3 ± 0.6	11.0 ± 1.0		20.5 ± 2.1^b^		9.3 ± 1.2	19.7 ± 6.4		9.3 ± 0.6		22.0 ± 4.2^b^		9.7 ± 0.6		22.0 ± 0.0		9.5 ± 0.7		26.0 ± 1.4^b^	
SZMC 25875	10.0 ± 0.0	35.7 ± 1.5	9.3 ± 1.5		25.0 ± 8.5^b^		10.3 ± 0.6	30.7 ± 1.5		11.7 ± 3.1		27.5 ± 0.7^b^		10.3 ± 0.6		26.3 ± 2.5		11.0 ± 0.0		26.0 ± 2.8^b^	

a No inhibition was observed, C is equal to IZ.

b Inhibition was observed in 2 replicates.

c Inhibition was observed in 1 replicate.

The data are presented as means of 3 replicates ± standard deviations, except the highlighted data.

### 3.2 Carbon source utilization profile

*P. resinovorans* SZMC 25872 as the most promising biocontrol isolate together with *A. tumefaciens* SZMC 14557 and *A. vitis* SZMC 21396, which proved to be the most susceptible pathogenic strains (Subsection 3.1) were selected for carbon source utilization assays. All strains were grown in LMM supplied with the different compounds (2 g/l) individually to test their ability to utilize them as the sole carbon source. The *P. resinovorans* SZMC 25872 (genotype I, Zhumakayev et al., 2021) strain showed substantial growth (OD_620_ >0.2) in the presence of 10 compounds (19, 39, 40, 42, 43, 47, 49, 63, 67, 68). OD_620_ values in the case of 9 substances (2, 6, 14, 17, 31, 37, 44, 46, 55) fell in the range of 0.1-0.2, and low growth (OD_620_ = 0.05-0.1) was observed in the presence of 10 compounds (7, 10, 12, 13, 16, 20, 34, 35, 45, 73). The strain was not able to utilize the remaining 45 substances as the sole carbon source.

*A. tumefaciens* SZMC 14557 utilized 17 compounds (2, 14, 21, 30, 36, 40, 43, 47, 56, 57, 58, 59, 61, 64, 65, 68, 69) with OD_620_ values above 0.2, 20 compounds (7, 8, 12, 15, 16, 17, 20, 22, 23, 25, 37, 38, 42, 50, 51, 63, 66, 67, 71, 74) with OD_620_ values in the range of 0.1-0.2, and 6 compounds (13, 35, 44, 52, 72, and 73) with low OD_620_ values (0.05-0.1). The remaining 31 compounds were not utilized as carbon source by this strain.

*A. vitis* SZMC 21396 grew in the presence of only 2 compounds (56 and 68) at the highest rate (OD_620_ >0.2), 18 substances (14, 15, 17, 20, 21, 22, 38, 42, 43, 47, 50, 58, 61, 64, 65, 66, 67,69) at moderate rate (OD_620_ 0.1-0.2), and 5 compounds (8, 30, 37, 59, 63) at the low rate (OD_620_ 0.05-0.1). The strain did not utilize the majority (49) of the tested 74 carbon-containing compounds

As only partial inhibition was observed in the case of *A. vitis* SZMC 21396 and this strain was able to utilize a relatively narrow carbon sources quantity among the tested 74 carbon-containing substances, further studies were not performed with the isolates belonging to this species. Among the 74 studied carbon sources, 11 compounds, viz. 2, 14, 17, 37, 40, 42, 43, 47, 63, 67, and 68, utilized sufficiently by both *P. resinovorans* SZMC 25872 and *A. tumefaciens* SZMC 14557, were selected to test the effect of carbon sources on the suppression of *A. tumefaciens* SZMC 14557 by *P. resinovorans* SZMC 25872 (Subsection 3.3).

### 3.3 The effect of carbon sources on the antagonistic potential of *P. resinovorans* against *A. tumefaciens*

The influence of different carbon sources on the suppression of *A. tumefaciens* SZMC 14557 by *P. resinovorans* SZMC 25872 was evaluated using 11 compounds, which were utilized as the sole carbon source by both the pathogenic and the glyphosate-tolerant strain in liquid cultures (Subsection 3.2). On solid media, *P. resinovorans* SZMC 25872 could grow in the presence of most of the tested compounds, except for 47 and 67 (**Table 4**). The growth of *A. tumefaciens* SZMC 14557 was substantially inhibited by *P. resinovorans* SZMC 25872 in the presence of all tested compounds, and the highest inhibition zones of 17.67, 20.00, 25.33, and 19.00 mm were observed on plates containing 17, 37, 63, and 68, respectively (**Table 4**).

**Table 4:** Colony diameter (C, mm) and colony + inhibition zone diameter (IZ, mm) caused by *P. resinovorans* SZMC 25872 on *A. tumefaciens* SZMC 14557 in the presence of different carbon sources (means of 3 replicates ± standard deviations).

Carbon source	C	IZ
PDA	9.00 ± 0.00	20.67 ± 1.15
2-keto-D-gluconic acid	7.67 ± 0.58	15.33 ± 1.53
D-fructose	8.33 ± 0.58	9.00 ± 7.81
D-glucose	8.67 ± 0.58	17.67 ± 0.58
L-alanine	9.33 ± 0.58	20.00 ± 4.58
L-asparagine	9.33 ± 0.58	17.00 ± 1.73
L-glutamic acid	9.67 ± 0.58	17.33 ± 3.79
L-glutamine	9.67 ± 0.58	16.33 ± 3.51
L-malic acid	0.00 ± 0.00	0.00 ± 0.00
sodium pyruvate	10.33 ± 0.58	25.33 ± 1.53
starch	0.00 ± 0.00	0.00 ± 0.00
succinic acid	9.00 ± 0.00	19.00 ± 1.00

Our studies have confirmed the importance of carbon sources in the biocontrol efficacy. Among the 11 tested carbon-containing compounds, the diameter of the inhibition zones caused by *P. resinovorans* SZMC 25872 in the colony of *A. tumefaciens* SZMC 14557 ranged between 9.0 (14) and 25.33 mm (63). Subsequently, the culture supernatants obtained from minimal medium amended with the carbon sources resulting in the highest degree of inhibition (namely, 63, 37, 68, and 17) as well as the other media applied in the biocontrol studies media (PDB, LB, and YEG) were selected for studying the biocontrol activity, to examine the mode of inhibition, and to identify the compounds responsible for the inhibitory effect (Subsections 3.4-3.8).

### 3.4 The inhibitory effect of the cell-free culture filtrates of *P. resinovorans* on the growth of *A. tumefaciens*

**Figure 2:**
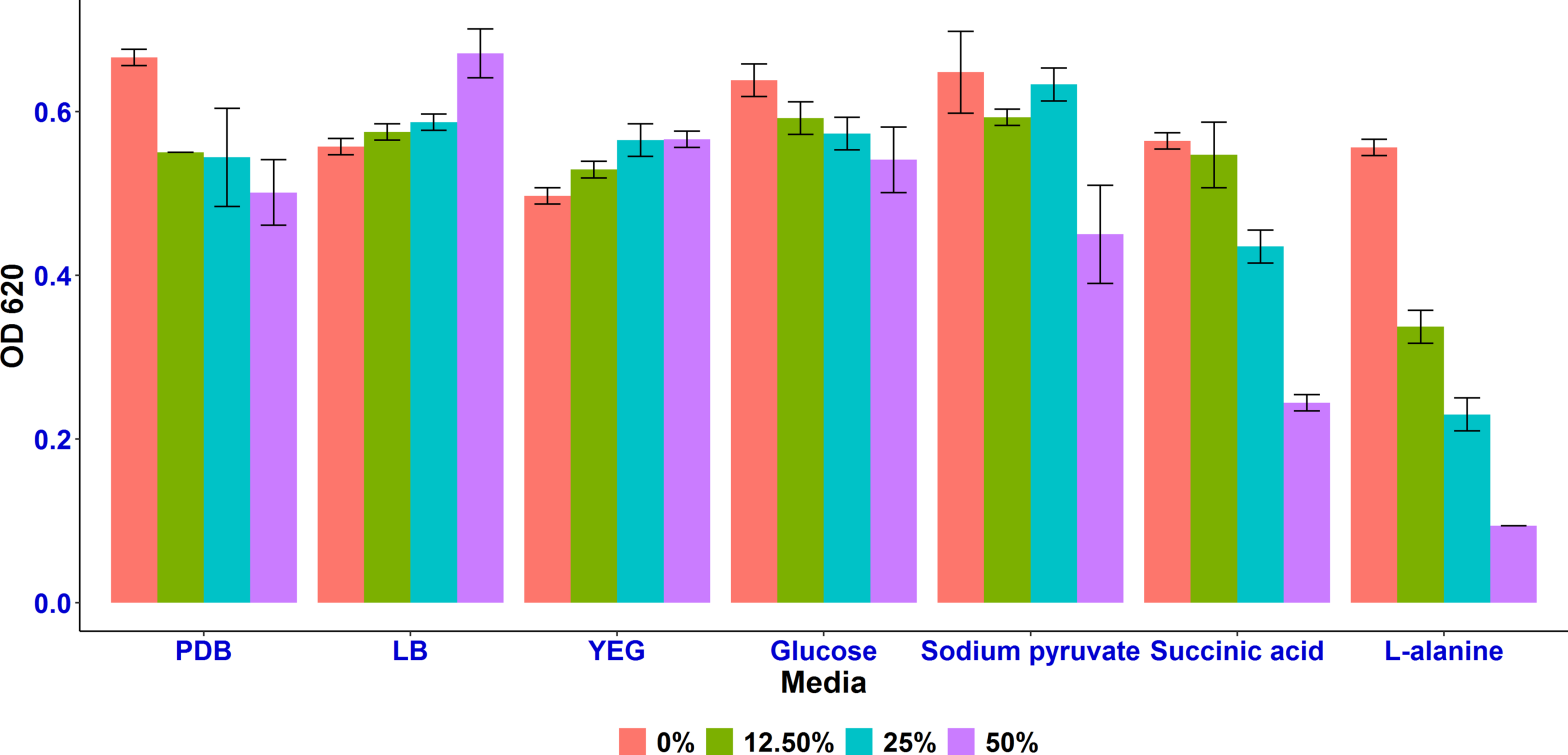
Cell density (OD_620_) of *A. tumefaciens* SZMC 14557 in the presence of 12.5, 25, and 50% concentration of CCF obtained from the culture supernatant of *P. resinovorans* SZMC 25872 grown in 7 media. 0% concentration of CCF is positive control (PDB without CCF).

Among the 7 different tested conditions, L-alanine and succinic acid as sole carbon sources were found to enhance the growth inhibition of *A. tumefaciens* SZMC 14557 by the selected *P. resinovorans* SZMC 25872 strain. Compared to the positive control, CCF samples obtained from LMM amended with L-alanine of *P. resinovorans* SZMC 25872 at 25% concentration resulted in more than 50% significant growth inhibition of the pathogenic strain (p<0.05), while increasing the concentration of CCFs to 50% led to a remarkable suppression of the *A. tumefaciens* SZMC 14557 strain (p<0.05) (**Figure 2**). Furthermore, the succinic acid-containing CCF samples at 25 and 50% also significantly inhibited the growth of *A. tumefaciens* SZMC 14557 (p<0.05), while in the case of CCFs obtained from LMM amended with sodium pyruvate significant difference from the positive control (p<0.05) was found only at 50% concentration. PDB-derived CCF also showed significant inhibition at 12.5-50% (p<0.05). No growth suppression was observed by the CCF samples of the examined *P. resinovorans* SZMC 25872 strain obtained from YEG, LB and glucose even at the highest tested concentration (**Figure 2**).

Based on the highest significant inhibition (p<0.05), L-alanine and succinic acid were found as the most promising carbon sources for the identification of the potential metabolites produced by *P. resinovorans* SZMC 25872 with antimicrobial activity against *A. tumefaciens* SZMC 14557. Moreover, all tested CCFs were subjected to HPLC-HRMS analysis (Subsection 3.8), as well as to the semi-quantitative determination of siderophore production (Subsection 3.5), and extracellular enzyme activity tests (Subsection 3.7). Being the filtrates showing the highest inhibitory effect on *A. tumefaciens* SZMC 14557, the CCF samples obtained from cultivation in LMM amended with L-alanine or succinic acid were selected for testing the hypothesis of siderophore and ROS-mediated antagonistic activity (Subsection 3.6).

Therefore, as CCFs of *P. resinovorans* SZMC 25872 obtained from minimal medium amended individually with L-alanine and succinic acid showed significant inhibition of *A. tumefaciens* SZMC 14557, the filtrates are proposed to contain inhibitory compounds. Furthermore, as *P. resinovorans* is presented here as a species with antagonistic potential towards *A. tumefaciens* for the first time, its CCF samples are expected to contain novel, yet unidentified bioactive metabolites, which are to be examined by HPLC-HRMS analysis (Subsection 3.8).

### 3.5 Siderophore production by *P. resinovorans* SZMC 25872 correlated with the growth inhibition of *A. tumefaciens*

The siderophore content of the CCF samples were analyzed by the CAS colorimetric method. The degree of inhibition caused by 25 and 50% CCF is presented in **Table 5** together with the data of siderophore production.

**Table 5:** Detection of siderophores (psu) produced by *P. resinovorans* SZMC 25872 in CCF samples, and the growth inhibition (%) of *A. tumefaciens* SZMC 14557 caused by 25 and 50% concentrations of CCF (means of 3 replicates ± standard deviations).

Media	Siderophore	Inhibition rate^*^	
	production (psu)	25% CCF	50% CCF
PDB	-43.73 ± 11.36	18.37 ± 8.79	24.77 ± 6.69
LB	-2.01 ± 3.72	-5.33 ± 1.67	-20.47 ± 5.15
Glucose	-15.63 ± 6.10	10.24 ± 3.00	15.15 ± 6.64
L-alanine	86.3 ± 1.69	58.69 ± 3.80	83.03 ± 0.37
Sodium pyruvate	78.97 ± 5.54	2.26 ± 3.77	30.56 ± 9.99
Succinic acid	80.41 ± 6.39	22.81 ± 3.99	56.80 ± 1.49
YEG	-64.81 ± 11.01	-13.68 ± 4.55	-13.82 ± 1.93

^*^The data of growth inhibition are presented as the decrease in the OD_620_ values of the treated samples in comparison with the positive control (PDB without CCF) calculated by the following formula: (Positive control − treated sample) × 100 ∕ positive control.

**Figure 3:**
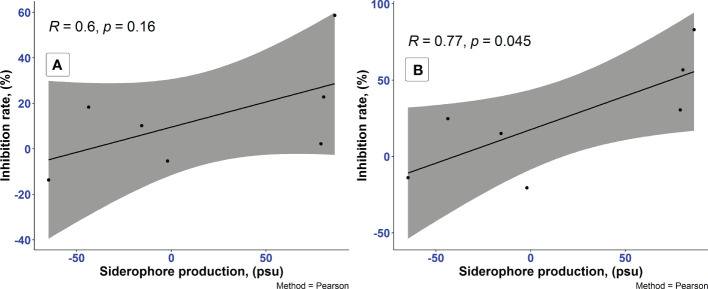
Analysis of correlation between the siderophore production of *P. resinovorans* SZMC 25872 and the growth inhibition of *A. tumefaciens* SZMC 14557 by 25% **(A)** and 50% **(B)** CCF (**Table 5**), Pearson coefficient. Grey color represents 95% confidence intervals.

In our studies, among 7 different media, the highest siderophore yields were detected in LMM amended with succinic acid followed by LMM with L-alanine and MM with sodium pyruvate, while no siderophore production was found in PDB, LB, YEG, and LMM supplied with glucose. Complex media, such as PDB, LB, and YEG contain certain amount of iron presumably sufficient for the *P. resinovorans* SZMC 25872 which resulted in negative values of siderophore production. LMM without iron sources stimulate the siderophore synthesis which confirmed with positive values in LMM amended with succinic acid, L-alanine, and sodium pyruvate. Importantly, the data presented in **Table 5** clearly shows the considerable inhibition of *A. tumefaciens* SZMC 14557 by samples with remarkable siderophore content. The highest degree of significant growth inhibition (p<0.05) due to CCFs of L-alanine or succinic acid-containing media might be associated with the siderophores detected in these samples. In contrast, no siderophores were found in PDB, LB, YEG, and LMM-glucose, and no significant inhibition of *A. tumefaciens* SZMC 14557 (p>0.05) by CCFs obtained from these media was observed either, except for the CCF of the *P. resinovorans* SZMC 25872 strain from PDB. The siderophore content and growth inhibition due to 25 and 50% concentrations of CCF from *P. resinovorans* SZMC 25872 showed insignificant (r = 0.6, p<0.16) and significant correlation (r = 0.77, p<0.05) for 25 and 50% CCF, respectively in the subsequent statistical analysis (Pearson correlation coefficient) (**Figure 3**).

Although considerable siderophore content was detected also in LMM supplied with sodium pyruvate, no remarkable inhibition was found in these CCF samples. It might be explained by the lower OD_630_ value of the reference sample (non-inoculated supernatant itself reacted well with CAS solution, and this reaction resulted in lower OD_630_, and consequently in higher values of siderophore percentage in the inoculated samples. However, significant inhibition (p<0.05) up to 30% was still observed in the samples treated with 50% CCF from sodium pyruvate-containing LMM (**Figure 2**). Remarkable siderophore production (86.30 and 80.41%) was observed in CCFs of *P. resinovorans* SZMC 25872 grown in LMM amended with L-alanine and succinic acid, respectively. Insignificant and significant correlations (r = 0.6, *r* ^2^ = 0.35, p<0.05; and r = 0.77, *r* ^2^ = 0.59, p<0.05) were found between these values and the growth inhibition of *A. tumefaciens* due to treatment with CCFs at 25 and 50% concentrations, respectively. Therefore, as siderophores are reported to be involved in the suppression of numerous pathogenic species (Kloepper et al., 1980; Lee et al., 2016; Tao et al., 2020), including *A. tumefaciens* (Penyalver et al., 2001), and as the enantio-pyochelin siderophore produced by the *P. protegens* CS1 strain showed the inhibitory effect against *Xanthomonas citri* ssp. *citri via* a ROS-mediated pathway (Michavila et al., 2017), siderophore-mediated suppression may be suggested as a potential mechanism involved in the growth inhibition of *A. tumefaciens* SZMC 14557. Therefore, the follow-up studies were aimed at the determination of the potential role of siderophores produced by *P. resinovorans* in the suppression of *A. tumefaciens* SZMC 14557 (Subsection 3.6).

### 3.6 The potential involvement of siderophores in the suppression of *A. tumefaciens*

**Figure 4:**
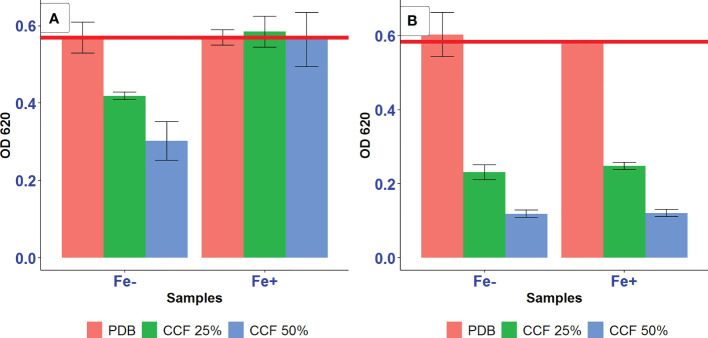
The effect of 25 and 50% CCF of *P. resinovorans* SZMC 25872 obtained from LMM amended with succinic acid **(A)** and L-alanine **(B)** on the growth of *A. tumefaciens* SZMC 14557 in the absence (Fe-) and presence (Fe+) of extra iron source (29 mg/l FeCl_3_·6H_2_O). PDB is positive control (no added CCF), CCF 25% and CCF 50% are the corresponding CCF concentrations. The red line represents the lowest value of the positive control.

The amendment of the culture media with additional iron and ascorbic acid were proposed to eliminate the action of siderophores as iron-removing or ROS-generating agents, respectively, to test the possibility of siderophore-mediated suppression of *A. tumefaciens* SZMC 14557 by *P. resinovorans* strain. In this assay, inhibition pattern was the same as described for testing the inhibitory effect of 12.5-50% CCF on the growth of *A. tumefaciens* SZMC 14557 (Subsection 3.4). The presence of CCF of *P. resinovorans* SZMC 25872 grown in L-alanine-containing LMM at either 25 or 50% also resulted in significant growth suppression compared to the positive control (p<0.05) (**Figure 4**). Moreover, the growth of *A. tumefaciens* SZMC 14557 treated with 25 and 50% CCF of SZMC 25872 from LMM-succinic acid was significantly inhibited (p<0.05) compared to the positive control. Interestingly, the addition of 29 mg/l FeCl_3_·6H_2_O to samples treated with the same CCF obtained from LMM-succinic acid caused the significantly increased growth of *A. tumefaciens* SZMC 14557 compared to the growth of the same samples without extra iron source (p<0.05), consequently, it was not significantly inhibited compared to the positive control (p>0.05). In addition, the extra iron source itself did not promote the growth of the positive control (no significant difference was found between the growth in pure PDB no containing no CCF with and without FeCl_3_·6H_2_O (p>0.05). Therefore, the significant stimulatory effect (p<0.05) of the extra iron on the bacterial growth was observed only when *A. tumefaciens* SZMC 14557 was treated with 25 and 50% CCF of *P. resinovorans* SZMC 25872 containing the highest amount of siderophores (**Table 5**, Subsection 3.5). Altogether, in the case of succinic acid-derived CCF, these results supported our hypothesis about siderophore-mediated suppression of *A. tumefaciens* SZMC 14557 by *P. resinovorans* SZMC 25872, because the inhibitory effect caused by CCF was eliminated by the presence of extra iron (**Figure 4**).

However, no significant increase in the growth of *A. tumefaciens* SZMC 14557 was observed between samples treated with 25-50% CCF of *P. resinovorans* SZMC 25872 obtained from LMM containing L-alanine due to the presence of an extra iron source (**Figure 4**). In comparison with the positive control, the CCF of the *P. resinovorans* SZMC 25872 strain applied at 25 and 50% resulted in a significant growth suppression of *A. tumefaciens* SZMC 14557 (p<0.05). The same inhibition pattern was found in all samples regardless of the presence of an additional iron source. These findings suggest different modes of inhibition caused by CCFs obtained from the culture supernatants of *P. resinovorans* SZMC 25872 grown in the presence of succinic acid and L-alanine. The inhibitory effect of CCF from LMM+succinic acid could be eliminated by the addition of 29 mg/l extra iron supporting the siderophore-mediated antagonism reported in several previous studies (Rane et al., 2008; Mishra and Arora, 2012; Sulochana et al., 2014; Michavila et al., 2017). The growth suppression by CCF from LMM+L-alanine remained unchanged in the presence of an additional iron, suggesting the production of different inhibitory compound(s) by *P. resinovorans* SZMC 25872 when grown in the presence of L-alanine as the sole carbon source.

The siderophores pyochelin and enantio-pyochelin produced by *P. aeruginosa* PAO1 and *P. protegens* CS1, respectively, were proposed to cause ROS generation leading to lipid peroxidation and the suppression of the plant pathogenic *X. citri* ssp. *citri via* a ROS-mediated pathway (Adler et al., 2012; Michavila et al., 2017). Enantio-pyochelin resulted in a significantly higher degree of ROS generation and lipid peroxidation in *X. citri* ssp. *citri*, but the addition of 1 mM ascorbic acid as a ROS-scavenging agent reduced the level of both parameters, which led to the elimination of growth suppression (Michavila et al., 2017). Based on these findings, the follow-up studies were performed to test the potential ROS-mediated nature of the suppression of *A. tumefaciens* SZMC 14557 by the CCF samples of *P. resinovorans* SZMC 25872. Specimens were treated with ascorbic acid to scavenge ROS possibly generated due to the presence of CCF, and thus to prevent *A. tumefaciens* SZMC 14557 from this oxidative stress factor.

**Figure 5:**
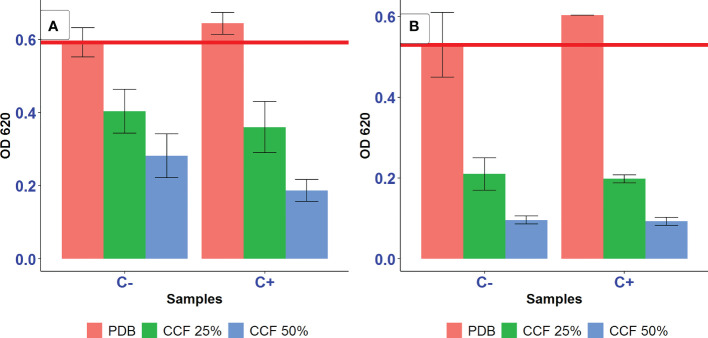
The effect of 25 and 50% CCF of *P. resinovorans* SZMC 25872 obtained from LMM amended with succinic acid **(A)** and L-alanine **(B)** on the growth of *A. tumefaciens* SZMC 14557 in the absence (C-) and presence (C+) of 0.17 mg/ml ascorbic acid. PDB is positive control (no added CCF), CCF 25% and CCF 50% are the corresponding CCF concentrations. The red line represents the lowest value of the positive control.

The addition of ascorbic acid did not promote the growth of the positive control significantly (p>0.05) (**Figure 5**). Furthermore, in the case of LMM+succinic acid, 25 and 50% CCF of *P. resinovorans* SZMC 25872 led to a significant suppression of *A. tumefaciens* SZMC 14557 compared to the positive control (p<0.05), regardless of the presence or absence of ascorbic acid. Ascorbic acid (vitamin C) also did not eliminate the significant, almost complete inhibition of *A. tumefaciens* SZMC 14557 (p<0.05) by CCF obtained from LMM+L-alanine either (**Figure 5**). The addition of 0.17 mg/ml ascorbic acid did not eliminate the growth inhibition of *A. tumefaciens* SZMC 14557 in samples treated with CCF of either LMM+succinic acid or L-alanine. Therefore, based on our findings, it can be suggested that the inhibition mode by CCF from either MM+succinic acid or MM+L-alanine does not include ROS generation.

### 3.7 Extracellular enzyme activity assays

**Figure 6:**
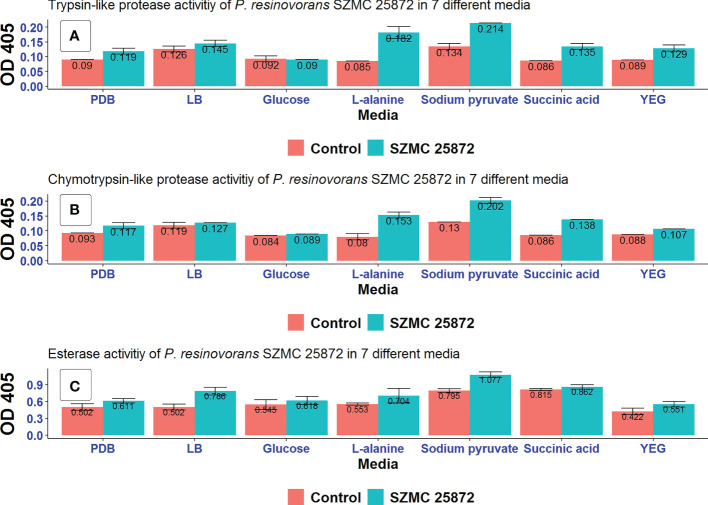
Extracellular enzyme activities **(A)** trypsin-like proteases, **(B)** chymotrypsin-like proteases, and **(C)** esterases of *P. resinovorans* SZMC 25872 grown in different media. Control is negative control (non-inoculated media).

Certain extracellular enzymes, such as chitinases, cellulases, proteases, and β-glucanases take part not only in the elimination of plant pathogens but can also indirectly stimulate plant growth (Mishra et al., 2020). Therefore, *P. resinovorans* SZMC 25872 was examined for its trypsin and chymotrypsin-like protease, as well as esterase activities in 7 different media (**Figure 6**).

High activity of all the 3 enzyme systems was observed in all examined media except for LMM amended with glucose. Among the studied carbon sources, sodium pyruvate and L-alanine stimulated the enzyme activities to the highest degree. No activity was detected in LMM amended with glucose. The highest trypsin- and chymotrypsin-like protease activities were found in LMM amended with sodium pyruvate and L-alanine as carbon source (**Figure 6**). Therefore, it can be suggested based on these results that extracellular protease activities might be involved in the inhibition of *A. tumefaciens* SZMC 14557, particularly in the case of LMM+L-alanine, as 25 and 50% CCF obtained from these media significantly inhibited *A. tumefaciens* SZMC 14557 (Subsections 3.4 and 3.6). Regarding esterases, activities could also be detected particularly when the strain was grown in LB and LMM+sodium pyruvate. Since the highest esterase activity was found in CCF of LB, and none of the CCFs obtained from this medium appeared to suppress *A. tumefaciens* SZMC 14557 remarkably (Subsection 3.4), it might be concluded that esterases do not play role in the inhibitory effect.

### 3.8 HPLC-HRMS analysis and identification of the bioactive metabolites potentially involved in the suppression of *A. tumefaciens*

According to the analysis of the HPLC-HRMS data, none of the known inhibitory agents was identified in the supernatant obtained from LMM amended with either succinic acid or L-alanine. However, several detected, potentially active metabolites are listed in **Table 6**.

**Table 6:** Identified metabolites with the potential to inhibit of *A. tumefaciens*.

Name	Molecular ion	Retention time, min	Molecular weight	Media
Compound 1	[M-H]^-^, [M+H]^+^	17.22	299.2458	AL
Compound 2	[M-H]^-^	7.68	337.0103	AL
Compound 3	[M-H]^-^	7.69	338.9682	AL
Compound 4^a^	[M+H]^+^	8.93	171.0898	SA
Compound 5^a^	[M+H]^+^	9.32	171.0898	SA
Compound 6^a^	[M+H]^+^	8.68	171.0898	SA
Compound 7^a^	[M+H]^+^	9.71	171.0898	SA
Compound 8^b^	[M+H]^+^	11.89	199.1208	SA
Compound 9^b^	[M+H]^+^	11.01	199.1208	SA
Compound 10^b^	[M+H]^+^	11.58	199.1208	SA
Compound 11^c^	[M+H]^+^	12.24	213.1365	SA
Compound 12^c^	[M+H]^+^	12.92	213.1365	SA
Compound 13	[M-H]^-^	5.96	275.1004	SA
Compound 14	[M-H]^-^	8.99	405.0480	SA
Compound 15	[M-H]^-^	8.98	374.1186	SA

a, b, c: According to their fragmentation pattern they belong to the same compound class. AL, L-alanine; SA, succinic acid.

The identification of the secondary metabolites was performed based on their exact mass. The exact mass of all the found metabolites was compared to those presented in 3 different secondary metabolite databases of the Department of Microbiology, University of Szeged (Hungary) involving all the published secondary metabolites of the *Pseudomonas* genus, as well as literature data.

Several compounds - such as (4, 5, 6, 7), (8, 9, 10) as well as (11 and 12) - detected in the supernatant of *P. resinovorans* SZMC 25872 grown in succinic medium - can be classified to the same chemical class as they show identical molecular weight (**Table 6**) and fragmentation pattern. Furthermore, all mentioned compounds have similar composition to homoserine lactones (N-butyryl-L-homoserine-lactone, N-hexanoyl-L-homoserine lactone, and N-heptanoyl-homoserine-lactone), respectively, but the *m/z* ratio of their main fragments was different from the known lactones, which possess 102.0555 *m/*z as the main fragment (Girard et al., 2017). Compounds 13, 14, and 15 also detected only in the supernatant recovered from medium amended with succinic acid as the sole carbon source might act as siderophore compounds, because the inhibitory effect by CCF obtained from this supernatant was eliminated in the presence of an extra iron source (Subsection 3.6).

**Figure 7:**
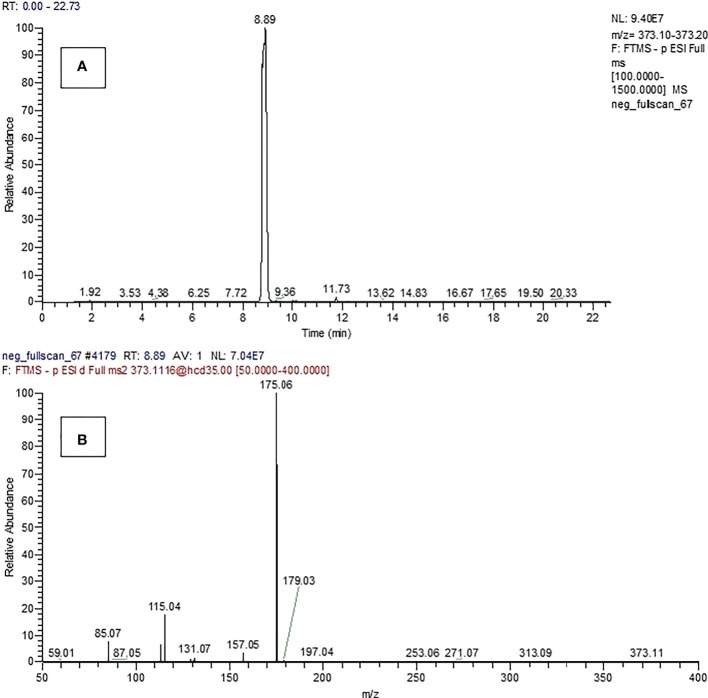
Selected ion chromatogram **(A)** and spectrum **(B)** of compound 15.

The selected ion chromatogram and spectrum of compound 15 produced by *P. resinovorans* SZMC 25872 was detected only when the strain was grown in LMM amended with L succinic acid (**Figure 7**).

## 4 Discussion

### 4.1 *In vitro* antagonistic activity of *P. resinovorans* towards *A. tumefaciens*

*P. resinovorans* SZMC 25872 showed the most sufficient inhibition activity towards most of the tested *A. tumefaciens* and *A. vitis* strains. To date, several bacterial species were reported to be capable of inhibiting *A. tumefaciens*. *P. fluorescens* B-4117 and Q8r1-96 suppressed the growth of both *A. tumefaciens* and *A. vitis* according to Dandurishvili et al. (2011). Molina et al. (2003) reported the inhibition of *A. tumefaciens via* the degradation of their acyl-homoserine lactones (AHL). The treatments of tomato plants with suspensions of both the vegetative cells and spores of *B. amyloliquefaciens* ssp. *plantarum* 32a resulted in a 79.1-87.5% reduction of the symptoms caused by the *A. tumefaciens* C58 and B6 isolates (Abdallah et al., 2018b). Metabolites, such as 1-(4-amino-2-hydroxyphenyl)ethanone produced by *P. liquidambari* as well as phenylacetic acid and behenic acid produced by *B. megaterium* L2 were found to inhibit the growth of *A. tumefaciens* in the studies of Zhou et al. (2020) and Xie et al. (2021), respectively.

In general, *Pseudomonas* and *Bacillus* genera are considered to contain the most promising species possessing antagonistic activity towards fungal and bacterial plant pathogens (Mnif and Ghribi, 2015). Phenazine-type 1-hydroxyphenazine produced by *P. aeruginosa* SD12 inhibited various fungal pathogens, such as *Alternaria alternata, A. solani, Bipolaris australiensis, Colletotrichum acutatum, Curvularia andropogonis, F. oxysporum, F. moniliforme, Pythium aphanidermatum*, and *Rhizoctonia solani in vitro* (Dharni et al., 2012). The *P. aeruginosa* LN strain was documented to possess biocontrol activity against *Xanthomonas axonopodis* pv. *malvacearum*, pv. *phaseoli* and pv. *citri* (Spago et al., 2014). *P. resinovorans* B11 was reported to possess biocontrol potential against plant pathogens *Pythium aphanidermatum* and *Monosporascus cannonballus* (Al−Daghari et al., 2019; Al−Daghari et al., 2020a). However, to the best of our knowledge, no strain of *P. resinovorans* species has been reported yet to possess the potential to antagonize *A. tumefaciens*. *E. adhaerens* was previously reported as a predator of *Micrococcus luteus* (Casida, 1982; Germida and Casida, 1983), but in our studies, *E. adhaerens* SZMC 25856 and *E. adhaerens* SZMC 25871 strains showed no inhibitory effect on any of the tested pathogens.

According to our findings, *P. resinovorans* SZMC 25872 can efficiently suppress the growth of *A. tumefaciens* strains, therefore, *P. resinovorans* can be proposed to broaden the spectrum of bacterial species with antagonistic activity.

### 4.2 Carbon source utilization profile

In the current literature, no data is available about the carbon source utilization profile of *P. resinovorans*. Therefore, the obtained findings could be compared to other reports only indirectly, by analyzing the composition of media which were used to grow these species in the corresponding references. The *P. resinovorans* SPR1 isolate was able to utilize eugenol as a source of energy by transforming it into vanillin, which broadens its biotechnological potential (Ashengroph et al., 2011). Another strain of *P. resinovorans*, CA10 was mentioned to be able to degrade carbazole through the utilization of this compound as carbon and nitrogen source (Shintani et al., 2013). *P*. *aeruginosa* GS-33 was found to utilize glucose, xylose, fructose, galactose, melibiose, mannose, glycerol, mannitol, xylitol, and malonate, but maltose, raffinose, trehalose, sucrose, inulin, sodium gluconate, salicin, dulcitol, inositol, erythritol, α-methyl-D-glucoside, rhamnose, cellobiose, melezitose, α-methyl D-mannoside, ONPG, D-arabinose and sorbose could not be utilized by the strain as carbon source (Patil et al., 2016).

### 4.3 The effect of carbon sources on the antagonistic potential of *P. resinovorans* against *A. tumefaciens*

The composition of the growth medium, especially the carbon source plays an important role in the antagonistic activity of microorganisms, particularly on the production of antibiotics. Glycerol as a sole carbon source suppressed the expression of the gene encoding for the 7-hydroxytropolone (pyoverdine-type siderophore), which is involved in the antimicrobial activity of *P. donghuensis* P482 (Matuszewska et al., 2021). In contrast, glycerol and glucose promoted the synthesis of the antibiotics 2,4-diacetylphloroglucinol, pyoluteorin, and pyochelin by *P. fluorescens* CHA0 (Duffy and Défago, 1999). The effect of carbon source might depend on the species of the potential biocontrol strain. The siderophores and antibiotics produced by *P. aeruginosa* D4 and *B. stratosphericus* FW3 inhibited the growth of a variety of bacterial plant pathogens, including *Burkholderia glumae* KACC 10 138*, Xanthomonas oxyzae* pv. *oryzae* KACC 10 208*, P. syringae* KACC 15 105*, Pectobacterium carotovorum* KACC 17 004 and *Ralstonia solanacearum* KACC 10 718. In terms of the tested carbon sources (lactose, sucrose, starch, and glucose), the inhibition pattern of the *P. aeruginosa* D4 strain was almost the same in the presence of lactose, sucrose, starch, and glucose, however, no inhibition of *R. solanacearum* was detected in the case of starch and glucose. In contrast, in the case of *B. stratosphericus*, the highest inhibition of *R. solanacearum* was found with starch, while *P. carotovorum* was not suppressed in the presence of lactose, and the inhibition of *B. gluma* and *P. syringae* was similar with all tested carbon sources (Durairaj et al., 2017).

### 4.4 The inhibitory effect of the cell-free culture filtrates of *P. resinovorans* on the growth of *A. tumefaciens*

The culture supernatants, different extracts, and filtrates of certain bacteria were previously reported to have inhibitory effect on phytopathogenic microorganisms. The benzene fraction obtained from the culture supernatant of *P. chlororaphis* ssp. *aureofaciens* DSM 6698 completely inhibited the growth of *R. solani*, *P. ultimum*, and *F. oxysporum* (Mezaache-Aichour et al., 2013). Different antimicrobial compounds, such as phenazines and siderophores were found to play role in the antagonistic effect of eleven isolates of fluorescent pseudomonads, including *P. fluorescens, P. aeruginosa, P. chlororaphis* ssp. *aurantiaca*, and *P. chlororaphis* ssp. *chlororaphis* (Shahid et al., 2021). Lee et al. (2016) reported about the inhibitory effect of the culture filtrates of a *P. aeruginosa* strain on the growth of *Corynebacterium glutamicum, B. subtilis, S. aureus*, and *A. tumefaciens*.

### 4.5 Siderophore production by *P. resinovorans* SZMC 25872 correlated with the growth inhibition of *A. tumefaciens*

The remarkable influence of the different growth media and carbon sources on the siderophore production of different microorganisms has been widely reported. Siderophores produced by the potential biocontrol agent of *Ganoderma boninense*, *Diaporthe miriciae* LF9 grown in PDB fell in the range of 32.81-76.05% in the presence and absence of heavy metals (Sim et al., 2020). Among different carbon sources, such as sucrose, glucose, fructose, Na-acetate, xylose, raffinose and maltose, sucrose was the most optimal carbon source to produce the siderophore rhodotorulic acid by the yeast *Rhodotorula mucilaginosa* ATCC 26423 (Andersen et al., 2003). Glycerol and mannose resulted in the highest and the lowest siderophore production by *B*. *megaterium* ATCC 19213, respectively, while no remarkable differences were observed among fructose, galactose, glucose, lactose, maltose, and sucrose (Santos et al., 2014). Glycerol itself did not result in a significant increase of the siderophore pyochelin by the *P. fluorescens* biocontrol strains. However, a slight increase was observed when glycerol was combined with diluted nutrient broth amended with yeast extract, and glucose gave a remarkable increase in the production of both pyochelin and salicylic acid (Duffy and Défago, 1999). Ghosh et al. (2015) reported that malt extract induced the highest production rate of siderophores by *P. aeruginosa*, casamino acids or succinic acid supported siderophore production, while no siderophores were detected in the medium containing peptone and beef extract. Payne (1994) has stated that the use of succinate instead of glucose as carbon source can result in enhanced siderophore production. Mendonca et al. (2020) reported that the siderophore secretion of different *Pseudomonas* strains (*P. putida* KT2440, *P. protegens* Pf-5, and *P. putida* S12) was promoted more by gluconeogenic than glycolytic substrates. Succinic acid, pH, and temperature had significant influence on siderophore synthesis by *P. aeruginosa* RZS9 (Shaikh et al., 2016). Glutamic acid, succinic acid, ornithine, alanine, aspartic acid, mannitol and lysine resulted in the largest halo zones indicating the siderophore-producing activity of *Burkholderia tropica* (Tenorio-Salgado et al., 2013).

### 4.6 The potential involvement of siderophores in the suppression of *A. tumefaciens*

Siderophore production is a well-known component of the suppression of plant pathogens by microbial biocontrol agents. Limiting the availability of iron leads to the restriction of microbial growth (O’Sullivan and O’Gara, 1992; Saha et al., 2016). Importantly, among the 3 types of siderophores, namely hydroxamates, catecholates, and carboxylates (Saha et al., 2016), the pathogenic and beneficial strains have to utilize different types, otherwise the siderophores produced by the biocontrol strain might be exploited by the pathogen, rather than being suppressed due to their action. The catecholate-type siderophore pyochelin produced by *P. aeruginosa* was effective only against bacteria with no ability to produce catecholate siderophores, while catecholate siderophore-producing bacteria were not affected by pyochelin (Adler et al., 2012). *A. tumefaciens* was reported to be a catecholate siderophore-producing species with its specific siderophore agrobactin (Leong and Neilands, 1981; Leong and Neilands, 1982; Sonoda et al., 2002). Furthermore, *A. tumefaciens* was able to promote its own growth by scavenging iron from catecholate-type siderophores produced by *Azotobacter vinelandii* to solubilize iron from insoluble iron salts (Page and Dale, 1986). Changes in color of halo zone on CAS agar plates from blue to purple or orange were attributed to the action of catechol or hydroxamate siderophores, respectively (Pérez-Miranda et al., 2007). Therefore, as our *P. resinovorans* SZMC 25872 strain produced orange halozones in the CAS agar assays (data not shown) they are likely to have produced hydroxamate-type siderophores, which cannot be exploited by *A. tumefaciens* SZMC 14557, resulting in its limited growth. The hydroxamate-type siderophore agrocin 434, produced by the *A. radiobacter* K84 strain, also inhibited pathogenic *A. tumefaciens* strains (Penyalver et al., 2001). It is obvious that the amount of siderophores is also a crucial factor in the siderophore-mediated suppression of pathogens. In our findings, the highest siderophore amount was detected in the CCF samples of the *P. resinovorans* SZMC 25872 strain grown in LMM containing succinic acid and L-alanine as the sole carbon source. Siderophores produced under these conditions are likely to have played a role in the inhibition of *A. tumefaciens* SZMC 14557. Based on the highest observed coefficient of determination (*r* ^2^), siderophores are suggested to account for more than half of the growth inhibition (59%), while the remaining 41% of suppression might have appeared due to different factors, such as further metabolites. Similarly to our results, the siderophore amount produced by *P. aeruginosa* KA19 was the highest in medium amended with succinate among 6 different carbon sources, including glucose, fructose, and mannitol. This strain significantly reduced black rot lesions caused by *Xanthomonas campestris* pv*. campestris* on *Brassica campestris*, compared to the untreated control (Mishra and Arora, 2012).

### 4.7 Extracellular enzyme activity assays

The secretion of lytic enzymes by rhizosphere-inhabiting microbes is known as an efficient response to combat phytopathogens *via* direct inhibition or competition for space and nutrients (Vörös et al., 2019; Mishra et al., 2020). Extracellular hydrolytic enzymes, such as chitinases, proteases, and β-1,3-glucanases produced by *Bacillus*, *Pseudomonas*, and *Trichoderma* strains were reported to have the inhibitory effect on a variety of plant pathogens, including *F. culmorum, F. oxysporum, F. solani, X. campestris* pv. *glycines*, and *R. solani* (Mishra et al., 2020). The antagonistic strains *B. amyloliquefaciens* SZMC 6161J and SZMC 6225J, along with *B. mojavensis* SZMC 6168J, and *B. subtilis* SZMC 6179J were reported to secrete chymotrypsin- and trypsin-like protease, as well as lipase enzymes (Vágvölgyi et al., 2013). Chitinases were also reported to trigger plant defense mechanisms and to have potential in the control of plant pathogenic fungi and insects (Gomaa, 2021).

### 4.8 HPLC-HRMS analysis and identification of the bioactive metabolites potentially involved in the suppression of *A. tumefaciens*

The genus *Pseudomonas* involves various species with the ability of producing different types of metabolites acting against a range of plant pathogens, some well-characterized examples are presented in **Supplementary Table 2**. By comparing the mass spectra of these compounds with those found in the NIST/Wiley database, these compounds 5-hydroxy-2-pentanone (RT: 4.25 min) and 4-hydroxy-4-methyl-2-pentanone (RT: 10.58 min) with molecular weights of 102.13 and 116.16, respectively, produced by *P. resinovorans* B11 were reported to inhibit *M. cannonballus* (Al−Daghari et al., 2020b). However, as *P. resinovorans* is a relatively new species possessing biocontrol potential, little information is available about its metabolites in the literature. The detected compounds might represent a novel class of inhibitory agents active against *A. tumefaciens*. Further investigations are required to verify exact metabolites and their inhibitory potential.

## Conclusion

In the present study, glyphosate-tolerant bacterial strains were examined for their potential ability to inhibit the growth of the plant pathogen *A. tumefaciens* and *A. vitis*. Both the living culture and the cell-free culture filtrates (CCF) of *P. resinovorans* SZMC 25872 could suppress *A. tumefaciens*. The highest growth inhibition was observed when succinic acid or L-alanine was applied as the sole carbon source. Based on our findings, siderophore-mediated suppression may be suggested as potential mode of action in the case of CCF obtained from succinic acid-containing medium, while the inhibitory effect of CCF from the medium supplemented with L-alanine might be attributed to the observed trypsin and chymotrypsin-like protease activities, as well as novel, yet unidentified metabolites. The HPLC-HRMS analysis of the CCF samples revealed the presence of several potential active metabolites, which might also play role in the inhibition of the plant pathogen.

Altogether, the remarkable amount of siderophores, activity of different extracellular enzymes, as well as potential bioactive metabolites make *P. resinovorans* SZMC 25872 a promising biocontrol candidate for utilization in agricultural applications. Further investigations are necessary to identify the metabolites produced in medium supplied with L-alanine or succinic acid as carbon sources.

## Data availability statement

The datasets presented in this study can be found in online repositories. The names of the repository/repositories and accession number(s) can be found below: https://www.ncbi.nlm.nih.gov/, MT955648. https://www.ncbi.nlm.nih.gov/, MT955649. https://www.ncbi.nlm.nih.gov/, MT955650. https://www.ncbi.nlm.nih.gov/, MT955645. https://www.ncbi.nlm.nih.gov/, MT955647. https://www.ncbi.nlm.nih.gov/, MT955646. https://www.ncbi.nlm.nih.gov/, MT955651.

## Author contributions

CV, LH, and TM designed the study and coordinated the draft of the manuscript. AZ wrote the first draft of the manuscript. MVa carried out HPLC-MS measurements and analyzed the obtained data. AS and TM verified the HPLC-MS measurements. AZ and MVö took part in the antagonistic assays and enzyme activity tests. SK performed the phylogenetic analyses. PR and TM contributed in the discussion of the manuscript. AZ, LH, and CV analyzed the results and designed the figures and tables. All authors commented on previous versions, read and approved the final manuscript. All authors contributed to the article and approved the submitted version.

## Funding

The research was supported by the Hungary-Serbia IPA Cross-border Co-operation Programme (PLANTSVITA; HUSRB/1602/41/0031). AZ was a grantee of the *Stipendium Hungaricum* Scholarship and *Stipendium Hungaricum* Dissertation Scholarship Programme (TEMPUS Foundation, Government of Hungary).

## Conflict of interest

The authors declare that the research was conducted in the absence of any commercial or financial relationships that could be construed as a potential conflict of interest.

## Publisher’s note

All claims expressed in this article are solely those of the authors and do not necessarily represent those of their affiliated organizations, or those of the publisher, the editors and the reviewers. Any product that may be evaluated in this article, or claim that may be made by its manufacturer, is not guaranteed or endorsed by the publisher.

## References

[B1] Abdallah D. B. Frikha-Gargouri O. Tounsi S. (2018b). Rhizospheric competence, plant growth promotion and biocontrol efficacy of *Bacillus amyloliquefaciens* subsp. *plantarum* strain 32a. Biol. Control 124, 61–67. doi: 10.1016/j.biocontrol.2018.01.013

[B2] Abdallah D. B. Tounsi S. Gharsallah H. Hammami A. Frikha-Gargouri O. (2018a). Lipopeptides from *Bacillus amyloliquefaciens* strain 32a as promising biocontrol compounds against the plant pathogen *Agrobacterium tumefaciens* . Environ. Sci. pollut. Res. 25, 36518–36529. doi: 10.1007/s11356-018-3570-1 30374716

[B3] Adler C. Corbalán N. S. Seyedsayamdost M. R. Pomares M. F. de Cristóbal R. E. Clardy J. . (2012). Catecholate siderophores protect bacteria from pyochelin toxicity. PLoS One 7, 1–7. doi: 10.1371/journal.pone.0046754 PMC3465284 23071628

[B4] Al-Daghari D. S. S. Al-Abri S. A. Al-Mahmooli I. H. Al-Sadi A. M. Velazhahan R. (2019). Efficacy of native antagonistic rhizobacteria in the biological control of *Pythium aphanidermatum* induced damping-off of cucumber in Oman. J. Plant Pathol. 102, 305–310. doi: 10.1007/s42161-019-00438-9

[B5] Al-Daghari D. S. S. Al-Mahmooli I. H. Al-Sadi A. M. AlSabahi J. N. Velazhahan R. (2020b). Production of antifungal metabolites by the antagonistic bacterial isolate *Pseudomonas resinovorans* B11. Ind. Phytopathol. 73, 771–775. doi: 10.1007/s42360-020-00264-5

[B6] Al-Daghari D. S. S. Al-Sadi A. M. Janke R. Al-Mahmooli I. H. Velazhahan R. (2020a). Potential of indigenous antagonistic rhizobacteria in the biological control of *Monosporascus* root rot and vine decline disease of muskmelon. Acta Agric. Scand. B Soil Plant Sci. 70, 371–380. doi: 10.1080/09064710.2020.1748703

[B7] Ambrosi C. Leoni L. Putignani L. Orsi N. Visca P. (2000). Pseudobactin biogenesis in the plant growth-promoting rhizobacterium *Pseudomonas* strain B10: identification and functional analysis of the l-ornithine N5-oxygenase (psbA) gene. J. Bacteriol. 182, 6233–6238. doi: 10.1128/JB.182.21.6233-6238.2000 11029447 PMC94761

[B8] Andersen D. Renshaw J. C. Wiebe M. G. (2003). Rhodotorulic acid production by *Rhodotorula mucilaginosa* . Mycol. Res. 107, 949–956. doi: 10.1017/S0953756203008220 14531617

[B9] Ashengroph M. Nahvi I. Zarkesh-Esfahani H. Momenbeik F. (2011). *Pseudomonas resinovorans* SPR1, a newly isolated strain with potential of transforming eugenol to vanillin and vanillic acid. N. Biotechnol. 28, 656–664. doi: 10.1016/j.nbt.2011.06.009 21689800

[B10] Benbrook C. M. (2016). Trends in glyphosate herbicide use in the United States and globally. Environ. Sci. Eur. 28, 1–15. doi: 10.1186/s12302-016-0070-0 27752438 PMC5044953

[B11] Bertani G. (1951). Studies on lysogenesis I: the mode of phage liberation by lysogenic *Escherichia coli* . J. Bacteriol. 62, 293–300. doi: 10.1128/jb.62.3.293-300.1951 14888646 PMC386127

[B12] Carvalho F. P. (2006). Agriculture, pesticides, food security and food safety. Environ. Sci. Policy 9, 685–692. doi: 10.1016/j.envsci.2006.08.002

[B13] Casida L. E. Jr (1982). *Ensifer adhaerens* gen. nov., sp. nov.: a bacterial predator of bacteria in soil. Int. J. Syst. Evol. Microbiol. 32, 339–345. doi: 10.1099/00207713-32-3-339

[B14] Chandler D. Bailey A. S. Mark Tatchell G. Davidson G. Greaves J. Grant W. P. (2011). The development, regulation and use of biopesticides for integrated pest management. Philos. Trans. R. Soc B Biol. Sci. 366, 1987–1998. doi: 10.1098/rstb.2010.0390 PMC3130386 21624919

[B15] Conner A. J. Dommisse E. M. (1992). Monocotyledonous plants as hosts for *Agrobacterium* . Int. J. Plant Sci. 153, 550–555. doi: 10.1086/297078

[B16] Dandurishvili N. Toklikishvili N. Ovadis M. Eliashvili P. Giorgobiani N. Keshelava R. . (2011). Broad-range antagonistic rhizobacteria *Pseudomonas fluorescens* and *Serratia plymuthica* suppress *Agrobacterium* crown gall tumours on tomato plants. J. Appl. Microbiol. 110, 341–352. doi: 10.1111/j.1365-2672.2010.04891.x 21091861

[B17] Dharni S. Alam M. Kalani K. Abdul-Khaliq A. K. Samad A. Srivastava S. K. . (2012). Production, purification, and characterization of antifungal metabolite from *Pseudomonas aeruginosa* SD12, a new strain obtained from tannery waste polluted soil. J. Microbiol. Biotechnol. 22, 674–683. doi: 10.4014/jmb.1109.09061 22561863

[B18] Druille M. García-Parisi P. A. Golluscio R. A. Cavagnaro F. P. Omacini M. (2016). Repeated annual glyphosate applications may impair beneficial soil microorganisms in temperate grassland. Agric. Ecosyst. Environ. 230, 184–190. doi: 10.1016/j.agee.2016.06.011

[B19] Duffy B. K. Défago G. (1999). Environmental factors modulating antibiotic and siderophore biosynthesis by *Pseudomonas fluorescens* biocontrol strains. Appl. Environ. Microbiol. 65, 2429–2438. doi: 10.1128/AEM.65.6.2429-2438.1999 10347023 PMC91358

[B20] Duke S. O. Powles S. B. (2008). Glyphosate: a once-in-a-century herbicide. Pest Manage. Sci. Former. Pestic. Sci. 64, 319–325. doi: 10.1002/ps.1518 18273882

[B21] Durairaj K. Velmurugan P. Park J. H. Chang W. S. Park Y. J. Senthilkumar P. . (2017). Potential for plant biocontrol activity of isolated *Pseudomonas aeruginosa* and *Bacillus stratosphericus* strains against bacterial pathogens acting through both induced plant resistance and direct antagonism. FEMS Microbiol. Lett. 364, 1–8. doi: 10.1093/femsle/fnx225 29069329

[B22] Filo A. Sabbatini P. Sundin G. W. Zabadal T. J. Safir G. R. Cousins P. S. (2013). Grapevine crown gall suppression using biological control and genetic engineering: A review of recent research. Am. J. Enol. Vitic. 64, 1–14. doi: 10.5344/ajev.2012.12038

[B23] Gaupp-Berghausen M. Hofer M. Rewald B. Zaller J. G. (2015). Glyphosate-based herbicides reduce the activity and reproduction of earthworms and lead to increased soil nutrient concentrations. Sci. Rep. 5, 1–9. doi: 10.1038/srep12886 PMC4542661 26243044

[B24] Germida J. J. Casida L. E. Jr (1983). *Ensifer adhaerens* predatory activity against other bacteria in soil, as monitored by indirect phage analysis. Appl. Environ. Microbiol. 45, 1380–1388. doi: 10.1128/aem.45.4.1380-1388.1983 16346275 PMC242466

[B25] Ghosh S. K. Pal S. Chakraborty N. (2015). The qualitative and quantitative assay of siderophore production by some microorganisms and effect of different media on its production. Int. J. Chem. Sci. 13, 1621–1629.

[B26] Girard L. Blanchet É. Intertaglia L. Baudart J. Stien D. Suzuki M. . (2017). Characterization of N-acyl homoserine lactones in *Vibrio tasmaniensis* LGP32 by a biosensor-based UHPLC-HRMS/MS method. Sensors (Switzerland) 17, 906. doi: 10.3390/s17040906 PMC5426830 28425948

[B27] Glare T. Caradus J. Gelernter W. Jackson T. Keyhani N. Köhl J. . (2012). Have biopesticides come of age? Trends Biotechnol. 30, 250–258. doi: 10.1016/j.tibtech.2012.01.003 22336383

[B28] Gomaa E. Z. (2021). Microbial chitinases: properties, enhancement and potential applications. Protoplasma. 258, 695–710. doi: 10.1007/s00709-021-01612-6 33483852

[B29] Hammami W. Labbé C. Chain F. Mimee B. Bélanger R. R. (2008). Nutritional regulation and kinetics of flocculosin synthesis by *Pseudozyma flocculosa* . Appl. Microbiol. Biotechnol. 80, 307–315. doi: 10.1007/s00253-008-1541-z 18542944

[B30] Hatvani L. Manczinger L. Marik T. Bajkán S. Vidács L. Bencsik O. . (2013). The complete degradation of acetanilide by a consortium of microbes isolated from river Maros. Acta Biol. Szeged. 57, 117–120.

[B31] He Y. Pantigoso H. A. Wu Z. Vivanco J. M. (2019). Co-inoculation of *Bacillus* sp. and *Pseudomonas putida* at different development stages acts as a biostimulant to promote growth, yield and nutrient uptake of tomato. J. Appl. Microbiol. 127, 196–207. doi: 10.1111/jam.14273 30955229

[B32] Hicks H. L. Comont D. Coutts S. R. Crook L. Hull R. Norris K. . (2018). The factors driving evolved herbicide resistance at a national scale. Nat. Ecol. Evol. 2, 163–176. doi: 10.1038/s41559-018-0470-1 29434350

[B33] Hoang D. T. Chernomor O. von Haeseler A. Minh B. Q. Vinh L. S. (2018). UFBoot2: Improving the ultrafast bootstrap approximation. Mol. Biol. Evol. 35, 518–522. doi: 10.1093/molbev/msx281 29077904 PMC5850222

[B34] Huber D. Cheng M. Winsor B. (2005). Association of severe *Corynespora* root rot of soybean with glyphosate-killed ragweed. Phytopathology 95, S45.

[B35] IARC (2015). “Some organophosphate insecticides and herbicides: diazinon, glyphosate, malathion, parathion, and tetrachlorvinphos,” in Monographs on the evaluation of carcinogenic risks to humans (International Agency for Research on Cancer ), 112.

[B36] Kalyaanamoorthy S. Minh B. Q. Wong T. K. F. von Haeseler A. Jermiin L. S. (2017). ModelFinder: fast model selection for accurate phylogenetic estimates. Nat. Methods 14, 587–589. doi: 10.1038/nmeth.4285 28481363 PMC5453245

[B37] Kassambara A. (2020) Gpubr: 'ggplot2' based publication ready plots. r package version 0.4.0. Available at: https://CRAN.R-project.org/package=ggpubr.

[B38] Katoh K. Standley D. M. (2013). MAFFT multiple sequence alignment software version 7: improvements in performance and usability. Mol. Biol. Evol. 30, 772–780. doi: 10.1093/molbev/mst010 23329690 PMC3603318

[B39] Kloepper J. W. Leong J. Teintze M. Schroth M. N. (1980). Enhanced plant growth by siderophores produced by plant growth-promoting rhizobacteria. Nature. 286, 885–886. doi: 10.1038/286885a0

[B40] Koller V. J. Fürhacker M. Nersesyan A. Mišík M. Eisenbauer M. Knasmueller S. (2012). Cytotoxic and DNA-damaging properties of glyphosate and roundup in human-derived buccal epithelial cells. Arch. Toxicol. 86, 805–813. doi: 10.1007/s00204-012-0804-8 22331240

[B41] Krüger M. Shehata A. A. Schrödl W. Rodloff A. (2013). Glyphosate suppresses the antagonistic effect of *Enterococcus* spp. on *Clostridium botulinum* . Anaerobe 20, 74–78. doi: 10.1016/j.anaerobe.2013.01.005 23396248

[B42] Lee Y. Kim Y. J. Lee J. H. Yu H. E. Lee K. Jin S. . (2016). TatC-dependent translocation of pyoverdine is responsible for the microbial growth suppression. J. Microbiol. 54, 122–130. doi: 10.1007/s12275-016-5542-9 26832668

[B43] Leong S. A. Neilands J. B. (1981). Relationship of siderophore-mediated iron assimilation to virulence in crown gall disease. J. Bacteriol. 147, 482–491. doi: 10.1128/jb.147.2.482-491.1981 6455414 PMC216068

[B44] Leong S. A. Neilands J. B. (1982). Siderophore production by phytopathogenic microbial species. Arch. Biochem. Biophys. 218, 351–359. doi: 10.1016/0003-9861(82)90356-3 6218783

[B45] Lioi M. B. Scarfi M. R. Santoro A. Barbieri R. Zeni O. Salvemini F. . (1998). Cytogenetic damage and induction of pro-oxidant state in human lymphocytes exposed *in vitro* to gliphosate, vinclozolin, atrazine, and DPX-E9636. Environ. Mol. Mutagen. 32, 39–46. doi: 10.1002/(SICI)1098-2280(1998)32:1<39::AID-EM5>3.0.CO;2-6 9707097

[B46] Matthysse A. G. (2005). The genus *Agrobacterium* . Prokaryotes 5, 91–114. doi: 10.1007/0-387-30745-1_5

[B47] Matuszewska M. Maciąg T. Rajewska M. Wierzbicka A. Jafra S. (2021). The carbon source-dependent pattern of antimicrobial activity and gene expression in *Pseudomonas donghuensis* P482. Sci. Rep. 11, 1–17. doi: 10.1038/s41598-021-90488-w 34040089 PMC8154892

[B48] Mendiburu F. (2020) Agricolae: Statistical procedures for agricultural research. r package version 1.3-3. Available at: https://CRAN.R-project.org/package=agricolae.

[B49] Mendonca C. M. Yoshitake S. Wei H. Werner A. Sasnow S. S. Thannhauser T. W. . (2020). Hierarchical routing in carbon metabolism favors iron-scavenging strategy in iron-deficient soil *Pseudomonas* species. Proc. Natl. Acad. Sci. 117, 32358–32369. doi: 10.1073/pnas.20163801 33273114 PMC7768705

[B50] Mezaache-Aichour S. Guechi A. Zerroug M. M. Nicklin J. Strange R. N. (2013). Antimicrobial activity of *Pseudomonas* secondary metabolites. Pharmacogn. Commun. 3, 39–44. doi: 10.5530/pc.2013.3.8

[B51] Michavila G. Adler C. De Gregorio P. R. Lami M. J. Caram Di Santo M. C. Zenoff A. M. . (2017). *Pseudomonas protegens* CS1 from the lemon phyllosphere as a candidate for citrus canker biocontrol agent. Plant Biol. 19, 608–617. doi: 10.1111/plb.12556 28194866

[B52] Mishra S. Arora N. K. (2012). Evaluation of rhizospheric *Pseudomonas* and *Bacillus* as biocontrol tool for *Xanthomonas campestris* pv *campestris* . World J. Microbiol. Biotechnol. 28, 693–702. doi: 10.1007/s11274-011-0865-5 22806865

[B53] Mishra P. Mishra J. Dwivedi S. K. Arora N. K. (2020). “Microbial enzymes in biocontrol of phytopathogens,” in Microbial enzymes: roles and applications in industries. microorganisms for sustainability, vol. 11 . Eds. Arora N. Mishra J. Mishra V. (Singapore: Springer), 259–285. doi: 10.1007/978-981-15-1710-5_10

[B54] Mnif I. Ghribi D. (2015). Potential of bacterial derived biopesticides in pest management. Crop Prot. 77, 52–64. doi: 10.1016/j.cropro.2015.07.017

[B55] Molina L. Constantinescu F. Michel L. Reimmann C. Duffy B. Défago G. (2003). Degradation of pathogen quorum-sensing molecules by soil bacteria: a preventive and curative biological control mechanism. FEMS Microbiol. Ecol. 45, 71–81. doi: 10.1016/S0168-6496(03)00125-9 19719608

[B56] Motta E. V. S. Raymann K. Moran N. A. (2018). Glyphosate perturbs the gut microbiota of honey bees. Proc. Natl. Acad. Sci. U.S.A. 115, 10305–10310. doi: 10.1073/pnas.1803880115 30249635 PMC6187125

[B57] Nguyen L. T. Schmidt H. A. von Haeseler A. Minh B. Q. (2015). IQ-TREE: A fast and effective stochastic algorithm for estimating maximum likelihood phylogenies. Mol. Biol. Evol. 32, 268–274. doi: 10.1093/molbev/msu300 25371430 PMC4271533

[B58] O’sullivan D. J. O’Gara F. (1992). Traits of fluorescent *Pseudomonas* spp. involved in suppression of plant root pathogens. Microbiol. Rev. 56, 662–676. doi: 10.1128/mr.56.4.662-676.1992 1480114 PMC372893

[B59] Page W. J. Dale P. L. (1986). Stimulation of *Agrobacterium tumefaciens* growth by *Azotobacter vinelandii* ferrisiderophores. Appl. Environ. Microbiol. 51, 451–454. doi: 10.1128/aem.51.2.451-454.1986 16347002 PMC238895

[B60] Pang Z. Raudonis R. Glick B. R. Lin T. J. Cheng Z. (2019). Antibiotic resistance in *Pseudomonas aeruginosa*: mechanisms and alternative therapeutic strategies. Biotechnol. Adv. 37, 177–192. doi: 10.1016/j.biotechadv.2018.11.013 30500353

[B61] Patil S. Paradeshi J. Chaudhari B. (2016). Suppression of charcoal rot in soybean by moderately halotolerant *Pseudomonas aeruginosa* GS-33 under saline conditions. J. Basic Microbiol. 56, 889–899. doi: 10.1002/jobm.201600008 27213894

[B62] Payne S. M. (1994). Detection, isolation, and characterization of siderophores. Methods Enzymol. 235, 329–344. doi: 10.1016/0076-6879(94)35151-1 8057905

[B63] Pedersen T. L. (2020) Patchwork: The composer of plots. r package version 1.1.1. Available at: https://CRAN.R-project.org/package=patchwork.

[B64] Penyalver R. Oger P. López M. M. Farrand S. K. (2001). Iron-binding compounds from *Agrobacterium* spp.: Biological control strain *Agrobacterium rhizogenes* K84 produces a hydroxamate siderophore. Appl. Environ. Microbiol. 67, 654–664. doi: 10.1128/AEM.67.2.654-664.2001 11157228 PMC92632

[B65] Pérez-Miranda S. Cabirol N. George-Téllez R. Zamudio-Rivera L. S. Fernández F. J. (2007). O-CAS, a fast and universal method for siderophore detection. J. Microbiol. Methods 70, 127–131. doi: 10.1016/j.mimet.2007.03.023 17507108

[B66] Rane M. R. Sarode P. D. Chaudhari B. L. Chincholkar S. B. (2008). Exploring antagonistic metabolites of established biocontrol agent of marine origin. Appl. Biochem. Biotechnol. 151, 665–675. doi: 10.1007/s12010-008-8288-y 18626581

[B67] Rueda-Ruzafa L. Cruz F. Roman P. Cardona D. (2019). Gut microbiota and neurological effects of glyphosate. Neurotoxicology 75, 1–8. doi: 10.1016/j.neuro.2019.08.006 31442459

[B68] Saha M. Sarkar S. Sarkar B. Sharma B. K. Bhattacharjee S. Tribedi P. (2016). Microbial siderophores and their potential applications: a review. Environ. Sci. pollut. Res. 23, 3984–3999. doi: 10.1007/s11356-015-4294-0 25758420

[B69] Santos S. Neto I. F. F. Machado M. D. Soares H. M. V. M. Soares E. V. (2014). Siderophore production by *Bacillus megaterium*: Effect of growth phase and cultural conditions. Appl. Biochem. Biotechnol. 172, 549–560. doi: 10.1007/s12010-013-0562-y 24101562

[B70] Schwyn B. Neilands J. B. (1987). Universal chemical assay for the detection and determination of siderophores. Anal. Biochem. 160, 47–56. doi: 10.1016/0003-2697(87)90612-9 2952030

[B71] Setlhare B. Kumar A. Mokoena M. P. Olaniran A. O. (2019). Catechol 1, 2-dioxygenase is an analogue of homogentisate 1, 2-dioxygenase in *Pseudomonas chlororaphis* strain UFB2. Int. J. Mol. Sci. 20, 61. doi: 10.3390/ijms20010061 PMC6337169 30586858

[B72] Shahid I. Han J. Hardie D. Baig D. N. Malik K. A. Borchers C. H. . (2021). Profiling of antimicrobial metabolites of plant growth promoting pseudomonas spp. isolated from different plant hosts. 3 Biotech. 11, 1–14. doi: 10.1007/s13205-020-02585-8 PMC7801537 33489669

[B73] Shaikh S. S. Wani S. J. Sayyed R. Z. (2016). Statistical-based optimization and scale-up of siderophore production process on laboratory bioreactor. 3 Biotech. 6, 1–10. doi: 10.1007/s13205-016-0365-2 PMC4754294 28330140

[B74] Shintani M. Hosoyama A. Ohji S. Tsuchikane K. Takarada H. Yamazoe A. . (2013). Complete genome sequence of the carbazole degrader *Pseudomonas resinovorans* strain CA10 (NBRC 106553). Genome Announc. 1, 2012–2013. doi: 10.1128/genomeA.00488-13 PMC3735057 23887915

[B75] Sim C. S. F. Cheow Y. L. Ng S. L. Ting A. S. Y. (2020). Can metal-tolerant endophytic biocontrol agents promote plant-growth under metal stress? Acta Biol. Szeged. 63, 169–179. doi: 10.14232/abs.2019.2.169-179

[B76] Sonoda H. Suzuki K. Yoshida K. (2002). Gene cluster for ferric iron uptake in *Agrobacterium tumefaciens* MAFF301001. Genes Genet. Syst. 77, 137–146. doi: 10.1266/ggs.77.137 12207035

[B77] Spago F. R. Ishii Mauro C. S. Oliveira A. G. Beranger J. P. O. Cely M. V. T. Stanganelli M. M. . (2014). *Pseudomonas aeruginosa* produces secondary metabolites that have biological activity against plant pathogenic *Xanthomonas* species. Crop Prot. 62, 46–54. doi: 10.1016/j.cropro.2014.04.011

[B78] Subramoni S. Nathoo N. Klimov E. Yuan Z. C. (2014). *Agrobacterium tumefaciens* responses to plant-derived signaling molecules. Front. Plant Sci. 5. doi: 10.3389/fpls.2014.00322 PMC4086400 25071805

[B79] Sulochana M. B. Jayachandra S. Y. Kumar S. K. A. Dayanand A. (2014). Antifungal attributes of siderophore produced by the *Pseudomonas aeruginosa* JAS-25. J. Basic Microbiol. 54, 418–424. doi: 10.1002/jobm.201200770 23686966

[B80] Syed Ab Rahman S. F. Singh E. Pieterse C. M. J. Schenk P. M. (2018). Emerging microbial biocontrol strategies for plant pathogens. Plant Sci. 267, 102–111. doi: 10.1016/j.plantsci.2017.11.012 29362088

[B81] Tao X. Zhang H. Gao M. Li M. Zhao T. Guan X. (2020). *Pseudomonas* species isolated *via* high-throughput screening significantly protect cotton plants against *Verticillium* wilt. AMB Express 10, 1–12. doi: 10.1186/s13568-020-01132-1 PMC7593376 33118043

[B82] Tarazona J. V. Court-Marques D. Tiramani M. Reich H. Pfeil R. Istace F. . (2017). Glyphosate toxicity and carcinogenicity: a review of the scientific basis of the European union assessment and its differences with IARC. Arch. Toxicol. 91, 2723–2743. doi: 10.1007/s00204-017-1962-5 28374158 PMC5515989

[B83] Tenorio-Salgado S. Tinoco R. Vazquez-Duhalt R. Caballero-Mellado J. Perez-Rueda E. (2013). Identification of volatile compounds produced by the bacterium *Burkholderia tropica* that inhibit the growth of fungal pathogens. Bioengineered 4, 236–243. doi: 10.4161/bioe.23808 23680857 PMC3728194

[B84] Thakore Y. (2006). The biopesticide market for global agricultural use. Ind. Biotechnol. 2, 194–208. doi: 10.1089/ind.2006.2.194

[B85] Vágvölgyi C. Magyar K. Papp T. Palágyi Z. Ferenczy L. Nagy Á. (1996). Value of substrate utilization data for characterization of *Mucor* isolates. Can. J. Microbiol. 42, 613–616. doi: 10.1139/m96-083

[B86] Vágvölgyi C. Sajben-Nagy E. Bóka B. Vörös M. Berki A. Palágyi A. . (2013). Isolation and characterization of antagonistic *Bacillus* strains capable to degrade ethylenethiourea. Curr. Microbiol. 66, 243–250. doi: 10.1007/s00284-012-0263-8 23143288

[B87] Van Bruggen A. H. C. He M. M. Shin K. Mai V. Jeong K. C. Finckh M. R. . (2018). Environmental and health effects of the herbicide glyphosate. Sci. Total Environ. 616–617, 255–268. doi: 10.1016/j.scitotenv.2017.10.309 29117584

[B88] Vörös M. Manczinger L. Kredics L. Szekeres A. Shine K. Alharbi N. S. . (2019). Influence of agro-environmental pollutants on a biocontrol strain of *Bacillus velezensis* . Microbiologyopen 8, e00660,1–12. doi: 10.1002/mbo3.660 29938920 PMC6436430

[B89] Wickham H. (2016). ggplot2: Elegant graphics for data analysis (Springer-Verlag New York: Springer Cham).

[B90] Wise A. A. Liu Z. Binns A. N. (2006). Culture and maintenance of *Agrobacterium* strains. Methods Mol. Biol. 343, 3–13. doi: 10.1385/1-59745-130-4:3 16988330

[B91] Xie Y. Peng Q. Ji Y. Xie A. Yang L. Mu S. . (2021). Isolation and identification of antibacterial bioactive compounds from *Bacillus megaterium* L2. Front. Microbiol. 12. doi: 10.3389/fmicb.2021.645484 PMC8024468 33841370

[B92] Xin X. F. Kvitko B. He S. Y. (2018). *Pseudomonas syringae*: What it takes to be a pathogen. Nat. Rev. Microbiol. 16, 316–328. doi: 10.1038/nrmicro.2018.17 29479077 PMC5972017

[B93] Zhou J. W. Jia A. Q. Tan X. J. Chen H. Sun B. Huang T. Z. . (2020). 1-(4-Amino-2-hydroxyphenyl)ethenone suppresses *Agrobacterium tumefaciens* virulence and metabolism. Front. Microbiol. 11. doi: 10.3389/fmicb.2020.584767 PMC7688917 33281779

[B94] Zhumakayev A. R. Vörös M. Szekeres A. Rakk D. Vágvölgyi C. Szűcs A. . (2021). Comprehensive characterization of stress tolerant bacteria with plant growth-promoting potential isolated from glyphosate-treated environment. World J. Microbiol. Biotechnol. 37, 1–17. doi: 10.1007/s11274-021-03065-8 33963474

