# Supplementary material for: Characterization of the antagonistic potential of the glyphosate-tolerant Pseudomonas resinovorans SZMC 25872 strain against the plant pathogenic bacterium Agrobacterium tumefaciens
Source: Front Plant Sci. 2022 Nov 28;13:1034237. doi: 10.3389/fpls.2022.1034237 (PMC9743988; doi:10.3389/fpls.2022.1034237)
Supplement: Supplementary file 1 [file DataSheet_1.docx]

Supplementary Material

# Supplementary Figures and Tables

## Supplementary Table 1. List of compounds tested as the sole carbon sources

| **№** | **Carbon source** |
| --- | --- |
| **1** | (-) quinic acid |
| **2** | 2-keto-D-gluconic acid |
| **3** | adenosine |
| **4** | alpha-methyl-D-mannoside |
| **5** | ascorbic acid |
| **6** | beta-alanine |
| **7** | beta-methyl-D-galactoside (1-O-methyl-beta-D-galactopyranoside) |
| **8** | cellobiose |
| **9** | cis-aconitic acid |
| **10** | cytidine |
| **11** | cytosine |
| **12** | D-arabinose |
| **13** | dextran |
| **14** | D-fructose |
| **15** | D-galactose |
| **16** | D-glucosamine |
| **17** | D-glucose |
| **18** | dihydroxyacetone |
| **19** | DL-isocitric acid |
| **20** | D-lyxose |
| **21** | D-mannitol |
| **22** | D-mannose |
| **23** | ethanol |
| **24** | fumaric acid |
| **25** | galactitol |
| **26** | gallic acid |
| **27** | gamma-butyrolactone |
| **28** | gentisic acid |
| **29** | gluconic acid |
| **30** | glycerol |
| **31** | glycine |
| **32** | i-erythritol |
| **33** | inosine |
| **34** | inulin |
| **35** | ketoisovaleric acid |
| **36** | lactose |
| **37** | L-alanine |
| **38** | L-arabinose |
| **39** | L-arginine |
| **40** | L-asparagine |
| **41** | L-citrulline |
| **42** | L-glutamic acid |
| **43** | L-glutamine |
| **44** | L-histidine |
| **45** | L-isoleucine |
| **46** | L-lysine |
| **47** | L-malic acid |
| **48** | L-methionine |
| **49** | L-ornithine |
| **50** | L-rhamnose |
| **51** | L-serine |
| **52** | L-sorbose |
| **53** | L-threonine |
| **54** | L-tryptophan |
| **55** | L-valine |
| **56** | maltose |
| **57** | melezitose |
| **58** | melibiose |
| **59** | myo-inositol |
| **60** | nicotinic acid |
| **61** | p-arbutin |
| **62** | protocatechuic acid (3,4-dixydroxybenzoic acid) |
| **63** | sodium pyruvate |
| **64** | raffinose (raffinose pentahydrate) |
| **65** | ribitol (adonitol) |
| **66** | sorbitol |
| **67** | starch |
| **68** | succinic acid |
| **69** | sucrose |
| **70** | thymine |
| **71** | uridine |
| **72** | vanillin |
| **73** | xylan |
| **74** | xylitol |

## Supplementary Table 2. *Pseudomonas* metabolites with antimicrobial activity against different plant pathogens

| **Antagonized organisms** | **Active compound** | **Producing strains** | **Reference** |
| --- | --- | --- | --- |
| **Phenazines** | | | |
| *Fusarium oxysporum* f. sp. *ciceris, Fusarium udum* | phenazine-1-carboxylic acid, oxychlororaphin | *P. aeruginosa* PNA1 | Anjaiah et al., 2003 |
| *Rhizoctonia solani*, *Xanthomonas oryzae* pv. *oryzae* | phenazine-1-carboxamide | *P. aeruginosa* MML2212 | Shanmugaiah et al., 2009 |
| *Macrophomina phaseolina,* *Dreschlera graminea* | phenazine | *P. aeruginosa* RM-3 | Minaxi and Saxena, 2010 |
| *Alternaria alternata, Alternaria solani, Backusella australiensis, Colletotrichum acutatum, Curvularia andropogonis, F. oxysporum, Fusarium moniliforme, Pythium aphanidermatum*, *R. solani* | 1-hydroxyphenazine | *P. aeruginosa* SD12 | Dharni et al., 2012 |
| *R. solani*, *Pythium ultimum,* *F. oxysporum* | phenazines | *P. chlororaphis* ssp. *aureofaciens* DSM 6698 | Mezaache-Aichour et al., 2013 |
| *M. phaseolina* | phenazine-1-carboxylic acid | *P. aeruginosa* GS-33 | Patil et al., 2016 |
| *R. solani* | phenazine like compound | *P. aeruginosa* VSMKU1 | Karmegham et al., 2020 |
| *Ganoderma boninense* | phenazine | *P. aeruginosa* UPMP3 | Parvin et al., 2020 |
| *Fusarium graminearum* | phenazine-1-carboxamide | *P. aeruginosa* NF011 | Sun et al., 2021 |
| **Siderophores** | | | |
| *Erwinia carotovora* | pseudobactin | *Pseudomonas* sp. B10 | Ambrosi et al., 2000 |
| *Xanthomonas campestris* pv. *campestris* | 4-hydroxy-2-nonyl-quinoline | *P. aeruginosa* KA19 | Mishra and Arora, 2012 |
| *F. oxysporum* f. sp. *ciceri*, *F. udum*, *Aspergillus niger* | siderophores (hydroxamate type) | *P. aeruginosa* JAS-25 | Sulochana et al., 2014 |
| *Xanthomonas citri* ssp. *citri* | enantio-pyochelin | *P. protegens* CS1 | Michavila et al., 2017 |
| *Verticillium dahliae* | 7-hydroxytropolone (7-HT) | *P. donghuensis 22G5* | Tao et al., 2020 |
| **Antibiotics and lipids** | | | |
| *F. oxysporum* f. sp. *cubense* | 2,4-DAPG | *P. aeruginosa* FP10 | Ayyadurai et al., 2006 |
| *Fusarium proliferatum* NCIM 1105, *A. niger* NCIM 596 | rhamnolipid | *P. aeruginosa* CPCL | Arutchelvi and Doble, 2010 |
| *F. oxysporum* f. sp. *radicis-cucumerinum* | 2,4-DAPG | *P. aeruginosa* P23 | Bradley and Punja, 2010 |
| *Pyricularia grisea* (TN508), *Gaeumannomyces graminis* (DSM1463), *F. oxysporum* (DSM62297), *X. campestris* (DSM3586), *Erwinia persicina* (HMGU155) | 2,4-DAPG | *P. putida,* *P. aeruginosa* | Sekar and Prabavathy, 2014 |
| *Fusarium verticillioides* | rhamnolipids | *P. aeruginosa* SS14 | Borah et al., 2016 |
| *M. phaseolina, F. oxysporum,* *Phytophthora nicotianae* | rhamnolipid | *P. aeruginosa* DR1 | Reddy et al., 2016 |
| *Rhizopus microsporus, F. oxysporum, A. niger, A. alternata, Penicillium digitatum* | pyrrolnitrin | *P. aeruginosa* PS24 | Uzair et al., 2018 |
| *Monosporascus cannonballus* | 4-hydroxy-4-methyl-2-pentanone | *P. resinovorans B11* | Al‑Daghari et al., 2020b |
| *F. oxysporum, F. moniliforme, R. solani, Colletotrichum gloeosporioides, Colletotrichum falcatum, A. niger*, *Aspergillus flavus* | iturin, surfactins, fengycin, diacetylphloroglucinol (DAPG), phenazine | *Pseudomonas* and *Bacillus* species | Ali et al., 2020 |
| *X. campestris, F. solani*, *Corticium invisium* | dirhamnolipid | *P. aeruginosa* RTE4 | Chopra et al., 2020 |
| **Other compounds** | | | |
| *M. phaseolina*, *Fusarium* sp., *Rhizoctonia* sp., *Alternaria* sp., *Aspergillus* sp. | 3,4-dihydroxy-N-methyl-4-(4-oxochroman-2-yl)butanamide | *P. aeruginosa* PGPR2 | Illakkiam et al., 2013 |
| *Erwinia amylovora* | L-2-amino-4-methoxy-*trans*-3-butenoic acid | *P. aeruginosa* | Lee et al., 2013 |
| *M. phaseolina* | purified exopolysaccharides | *P. aeruginosa* PF23 | Tewari and Arora, 2014 |
| Phytopathogenic *Xanthomonas* and *Pseudomonas* strains | tailocins | *P. fluorescens* SF4c | Fernandez et al., 2017 |
| *Phytophthora palmivora* | partially purified proteins | *P. aeruginosa* RS1 | Sowanpreecha and Rerngsamran, 2018 |
| *M. phaseolina* | 7-hydroxytropolone (7HT) | *P. donghuensis* SVBP6 | Muzio et al., 2020 |
| *Sporisorium scitamineum, Ceratocystis paradoxa, F. verticillioides* | hydrogen cyanide, salicylate, chitinase activity, exopolysaccharides | *P. aeruginosa* B18 | Singh et al., 2021 |

**References**

Al-Daghari, D. S. S., Al-Mahmooli, I. H., Al-Sadi, A. M., Al‑Sabahi, J. N., Velazhahan, R. (2020b). Production of antifungal metabolites by the antagonistic bacterial isolate *Pseudomonas resinovorans* B11. *Ind. Phytopathol.* 73, 771–775. doi: 10.1007/s42360-020-00264-5

Ali, S., Hameed, S., Shahid, M., Iqbal, M., Lazarovits, G., and Imran, A. (2020). Functional characterization of potential PGPR exhibiting broad-spectrum antifungal activity. *Microbiol. Res.* 232, 126389. doi: 10.1016/j.micres.2019.126389

Ambrosi, C., Leoni, L., Putignani, L., Orsi, N., and Visca, P. (2000). Pseudobactin biogenesis in the plant growth-promoting rhizobacterium *Pseudomonas* strain B10: identification and functional analysis of the L-ornithine N5-oxygenase (psbA) gene. *J. Bacteriol.* 182, 6233–6238. doi: 10.1128/JB.182.21.6233-6238.2000.

Anjaiah, V., Cornelis, P., and Koedam, N. (2003). Effect of genotype and root colonization in biological control of fusarium wilts in pigeonpea and chickpea by *Pseudomonas aeruginosa* PNA1. *Can. J. Microbiol.* 49, 85–91. doi: 10.1139/w03-011.

Arutchelvi, J., and Doble, M. (2010). Characterization of glycolipid biosurfactant from *Pseudomonas aeruginosa* CPCL isolated from petroleum-contaminated soil. *Lett. Appl. Microbiol.* 51, 75–82. doi: 10.1111/j.1472-765X.2010.02858.x.

Ayyadurai, N., Ravindra Naik, P., Sreehari Rao, M., Sunish Kumar, R., Samrat, S. K., Manohar, M., et al. (2006). Isolation and characterization of a novel banana rhizosphere bacterium as fungal antagonist and microbial adjuvant in micropropagation of banana. *J. Appl. Microbiol.* 100, 926–937. doi: 10.1111/j.1365-2672.2006.02863.x.

Borah, S. N., Goswami, D., Sarma, H. K., Cameotra, S. S., and Deka, S. (2016). Rhamnolipid biosurfactant against *Fusarium verticillioides* to control stalk and ear rot disease of maize. *Front. Microbiol.* 7, 1–10. doi: 10.3389/fmicb.2016.01505.

Bradley, G. G., and Punja, Z. K. (2010). Composts containing fluorescent pseudomonads suppress fusarium root and stem rot development on greenhouse cucumber. *Can. J. Microbiol.* 56, 896–905. doi: 10.1139/W10-076.

Chopra, A., Bobate, S., Rahi, P., Banpurkar, A., Mazumder, P. B., and Satpute, S. (2020). *Pseudomonas aeruginosa* RTE4: A tea rhizobacterium with potential for plant growth promotion and biosurfactant production. *Front. Bioeng. Biotechnol.* 8, 1–14. doi: 10.3389/fbioe.2020.00861.

Dharni, S., Alam, M., Kalani, K., Abdul-Khaliq, A. K., Samad, A., Srivastava, S. K., et al. (2012). Production, purification, and characterization of antifungal metabolite from *Pseudomonas aeruginosa* SD12, a new strain obtained from tannery waste polluted soil. *J. Microbiol. Biotechnol.* 22, 674–683. doi: 10.4014/jmb.1109.09061.

Fernandez, M., Godino, A., Príncipe, A., Morales, G. M., and Fischer, S. (2017). Effect of a *Pseudomonas fluorescens* tailocin against phytopathogenic *Xanthomonas* observed by atomic force microscopy. *J. Biotechnol.* 256, 13–20. doi: 10.1016/j.jbiotec.2017.07.002.

Illakkiam, D., Ponraj, P., Shankar, M., Muthusubramanian, S., Rajendhran, J., and Gunasekaran, P. (2013). Identification and structure elucidation of a novel antifungal compound produced by *Pseudomonas aeruginosa* PGPR2 against *Macrophomina phaseolina*. *Appl. Biochem. Biotechnol.* 171, 2176–2185. doi: 10.1007/s12010-013-0469-7.

Karmegham, N., Vellasamy, S., Natesan, B., Sharma, M. P., Al Farraj, D. A., and Elshikh, M. S. (2020). Characterization of antifungal metabolite phenazine from rice rhizosphere fluorescent pseudomonads (FPs) and their effect on sheath blight of rice. *Saudi J. Biol. Sci.* 27, 3313–3326. doi: 10.1016/j.sjbs.2020.10.007.

Lee, X., Azevedo, M. D., Armstrong, D. J., Banowetz, G. M., and Reimmann, C. (2013). The *Pseudomonas aeruginosa* antimetabolite L-2-amino-4-methoxy-trans-3-butenoic acid inhibits growth of *Erwinia amylovora* and acts as a seed germination-arrest factor. *Environ. Microbiol. Rep.* 5, 83–89. doi: 10.1111/j.1758-2229.2012.00395.x.

Mezaache-Aichour, S., Guechi, A., Zerroug, M. M., Nicklin, J., and Strange, R. N. (2013). Antimicrobial activity of *Pseudomonas* secondary metabolites. *Pharmacogn. Commun.* 3, 39–44. doi: 10.5530/pc.2013.3.8.

Michavila, G., Adler, C., De Gregorio, P. R., Lami, M. J., Caram Di Santo, M. C., Zenoff, A. M., et al. (2017). *Pseudomonas protegens* CS1 from the lemon phyllosphere as a candidate for citrus canker biocontrol agent. *Plant Biol.* 19, 608–617. doi: 10.1111/plb.12556

Minaxi and Saxena, J. (2010). Characterization of *Pseudomonas aeruginosa* RM-3 as a potential biocontrol agent. *Myco. Path.* 170, 181–193. doi: 10.1007/s11046-010-9307-4.

Mishra, S., and Arora, N. K. (2012). Evaluation of rhizospheric *Pseudomonas* and *Bacillus* as biocontrol tool for *Xanthomonas campestris* pv *campestris*. *World J. Microbiol. Biotechnol.* 28, 693–702. doi: 10.1007/s11274-011-0865-5.

Muzio, F. M., Agaras, B. C., Masi, M., Tuzi, A., Evidente, A., and Valverde, C. (2020). 7-hydroxytropolone is the main metabolite responsible for the fungal antagonism of *Pseudomonas donghuensis* strain SVBP6. *Environ. Microbiol.* 22, 2550–2563. doi: 10.1111/1462-2920.14925.

Parvin, W., Govender, N., Othman, R., Jaafar, H., Rahman, M., and Wong, M. Y. (2020). Phenazine from *Pseudomonas aeruginosa* UPMP3 induced the host resistance in oil palm (*Elaeis guineensis* Jacq.)-*Ganoderma boninense* pathosystem. *Sci. Rep.* 10, 1–12. doi: 10.1038/s41598-020-72156-7.

Patil, S., Paradeshi, J., and Chaudhari, B. (2016). Suppression of charcoal rot in soybean by moderately halotolerant *Pseudomonas aeruginosa* GS-33 under saline conditions. *J. Basic Microbiol.* 56, 889–899. doi: 10.1002/jobm.201600008.

Reddy, K. S., Khan, M. Y., Archana, K., Reddy, M. G., and Hameeda, B. (2016). Utilization of mango kernel oil for the rhamnolipid production by *Pseudomonas aeruginosa* DR1 towards its application as biocontrol agent. *Bioresour. Technol.* 221, 291–299. doi: 10.1016/j.biortech.2016.09.041.

Sekar, J., and Prabavathy, V. R. (2014). Novel Phl-producing genotypes of finger millet rhizosphere associated pseudomonads and assessment of their functional and genetic diversity. *FEMS Microbiol. Ecol.* 89, 32–46. doi: 10.1111/1574-6941.12354.

Shanmugaiah, V., Mathivanan, N., and Varghese, B. (2009). Purification, crystal structure and antimicrobial activity of phenazine-1-carboxamide produced by a growth-promoting biocontrol bacterium, *Pseudomonas aeruginosa* MML2212. *J. Appl. Microbiol.* 108, 703–711. doi: 10.1111/j.1365-2672.2009.04466.x.

Singh, P., Singh, R. K., Guo, D. J., Sharma, A., Singh, R. N., Li, D. P., et al. (2021). Whole genome analysis of sugarcane root-associated endophyte *Pseudomonas aeruginosa* B18—a plant growth-promoting bacterium with antagonistic potential against *Sporisorium scitamineum*. *Front. Microbiol.* 12, 1–21. doi: 10.3389/fmicb.2021.628376.

Sowanpreecha, R., and Rerngsamran, P. (2018). Biocontrol of orchid-pathogenic mold, *Phytophthora palmivora*, by antifungal proteins from *Pseudomonas aeruginosa* RS1. *Mycobiology* 46, 129–137. doi: 10.1080/12298093.2018.1468055.

Sulochana, M. B., Jayachandra, S. Y., Kumar, S. K. A., and Dayanand, A. (2014). Antifungal attributes of siderophore produced by the *Pseudomonas aeruginosa* JAS-25. *J. Basic Microbiol.* 54, 418–424. doi: 10.1002/jobm.201200770.

Sun, X., Xu, Y., Chen, L., Jin, X., and Ni, H. (2021). The salt-tolerant phenazine-1-carboxamide-producing bacterium *Pseudomonas aeruginosa* NF011 isolated from wheat rhizosphere soil in dry farmland with antagonism against *Fusarium graminearum*. *Microbiol. Res.* 245, 126673. doi: 10.1016/j.micres.2020.126673.

Tao, X., Zhang, H., Gao, M., Li, M., Zhao, T., and Guan, X. (2020). *Pseudomonas* species isolated via high-throughput screening significantly protect cotton plants against *Verticillium* wilt. *AMB Express* 10, 1–12. doi: 10.1186/s13568-020-01132-1.

Tewari, S., and Arora, N. K. (2014). Multifunctional exopolysaccharides from *Pseudomonas aeruginosa* PF23 involved in plant growth stimulation, biocontrol and stress amelioration in sunflower under saline conditions. *Curr. Microbiol.* 69, 484–494. doi: 10.1007/s00284-014-0612-x.

Uzair, B., Kausar, R., Bano, S. A., Fatima, S., Badshah, M., Habiba, U., et al. (2018). Isolation and molecular characterization of a model antagonistic *Pseudomonas aeruginosa* divulging *in vitro* plant growth promoting characteristics. *Biomed Res. Int.* 2018. doi: 10.1155/2018/6147380.
